# Comprehensive profiling of anaesthetised brain dynamics across phylogeny

**DOI:** 10.1101/2025.03.22.644729

**Published:** 2025-03-24

**Authors:** Andrea I. Luppi, Lynn Uhrig, Jordy Tasserie, Golia Shafiei, Kanako Muta, Junichi Hata, Hideyuki Okano, Daniel Golkowski, Andreas Ranft, Rudiger Ilg, Denis Jordan, Silvia Gini, Zhen-Qi Liu, Yohan Yee, Camilo M. Signorelli, Rodrigo Cofre, Alain Destexhe, David K. Menon, Emmanuel A. Stamatakis, Christopher W. Connor, Alessandro Gozzi, Ben D. Fulcher, Bechir Jarraya, Bratislav Misic

**Affiliations:** 1 Montréal Neurological Institute, McGill University, Montréal, QC, Canada; 2 Centre for Eudaimonia and Human Flourishing, Department of Psychiatry, University of Oxford, Oxford, UK; 3 St John’s College, University of Cambridge, Cambridge, UK; 4 Cognitive Neuroimaging Unit, CEA, INSERM, Université Paris-Saclay, NeuroSpin Center, Gif-sur-Yvette, France; 5 Department of Anesthesiology and Critical Care, Necker Hospital, Université de Paris Cité, Paris, France; 6 Center for Brain Circuit Therapeutics, Brigham and Women’s Hospital, Harvard Medical School, Boston, MA, USA; 7 Department of Psychiatry, Perelman School of Medicine, University of Pennsylvania, Philadelphia, PA, USA; 8 Graduate School of Human Health Sciences, Tokyo Metropolitan University, Arakawa, Tokyo, Japan; 9 Laboratory for Marmoset Neural Architecture, Center for Brain Science, RIKEN, Wako, Saitama Japan; 10 Department of Physiology, Keio University School of Medicine, Shinjuku, Tokyo, Japan; 11 Department of Neurology, Klinikum rechts der Isar, Technical University Munich, Munich, Germany; 12 Department of Anesthesiology and Intensive Care, Technical University of Munich, Munich, Germany.; 13 Asklepios Clinic, Department of Neurology, Bad Tolz, Germany; 14 Department of Anaesthesiology and Intensive Care Medicine, Klinikum rechts der Isar, Technical University Munich, Munich, Germany; 15 University of Applied Sciences and Arts Northwestern Switzerland, Muttenz, Switzerland; 16 Center for Neuroscience and Cognitive Systems, Istituto Italiano di Tecnologia, Rovereto, Italy; 17 Centre for Mind/Brain Sciences, University of Trento, Italy; 18 Center for Philosophy of Artificial Intelligence, University of Copenhagen, Copenhagen, Denmark; 19 Paris-Saclay University, CNRS, Paris-Saclay Institute for Neuroscience (NeuroPSI), Saclay, France; 20 Division of Anaesthesia, University of Cambridge, Cambridge, UK; 21 Department of Clinical Neurosciences, University of Cambridge, Cambridge, UK; 22 Department of Anesthesiology, Perioperative and Pain Medicine, Brigham and Women’s Hospital, Boston, MA, USA; 23 Department of Biomedical Engineering, Physiology and Biophysics, Boston University, Boston, Massachusetts; 24 School of Physics, The University of Sydney, Sydney, Australia; 25 Department of Neurology, Foch Hospital, Suresnes, France

**Keywords:** dynamics, anaesthesia, highlycomparative time-series analysis, cross-species neuroscience, gene expression, intrinsic timescales, computational modelling, deep-brain stimulation, functional MRI, calcium imaging

## Abstract

The intrinsic dynamics of neuronal circuits shape information processing and cognitive function. Combining non-invasive neuroimaging with anaesthetic-induced suppression of information processing provides a unique opportunity to understand how local dynamics mediate the link between neurobiology and the organism’s functional repertoire. To address this question, we compile a unique dataset of multi-scale neural activity during wakefulness and anesthesia encompassing human, macaque, marmoset, mouse and nematode. We then apply massive feature extraction to comprehensively characterize local neural dynamics across > 6 000 time-series features. Using dynamics as a common space for comparison across species, we identify a phylogenetically conserved dynamical profile of anaesthesia that encompasses multiple features, including reductions in intrinsic timescales. This dynamical signature has an evolutionarily conserved spatial layout, covarying with transcriptional profiles of excitatory and inhibitory neurotransmission across human, macaque and mouse cortex. At the network level, anesthetic-induced changes in local dynamics manifest as reductions in inter-regional synchrony. This relationship between local dynamics and global connectivity can be recapitulated *in silico* using a connectome-based computational model. Finally, this dynamical regime of anaesthesia is experimentally reversed *in vivo* by deep-brain stimulation of the centromedian thalamus in the macaque, resulting in restored arousal and behavioural responsiveness. Altogether, comprehensive dynamical phenotyping reveals that spatiotemporal isolation of local neural activity during anesthesia is conserved across species and anesthetics.

## INTRODUCTION

From invertebrates to primates, an essential function of the nervous system is to enable the organism to respond to an ever-changing environment. Indeed, brain activity is in constant flux—reflected in rich neural dynamics. Local signaling events then propagate via axonal projections, manifesting as coherent and patterned neural activity over the cortex. As a result, these dynamics span multiple spatial and temporal scales (1–5). Mapping how dynamics support brain function is a key goal in the neurosciences.

A prominent paradigm for understanding neural dynamics and their function is to manipulate them via general anaesthesia. Anaesthetic agents modulate neuronal signaling, altering local and global dynamics, reversibly suppressing the brain’s ability to process information and respond to the external environment. Neural changes observed at the macroscale can then be related to downstream effects on cognition and behaviour and upstream molecular mechanisms at the microscale. Although different species have evolved unique ways to respond to their specific environments, the behavioural effects of many anaesthetics are highly conserved across humans and other species, from primates to nematode worms (6–8), hinting at shared and fundamental underlying mechanisms. Systematically and reversibly perturbing brain function while recording neural activity provides a unique opportunity to understand how local dynamics mediate the link between anatomy and the organism’s functional repertoire.

Indeed, numerous studies have reported evidence of changes in neural activity that mirror transitions between wakefulness and anesthesia (6, 7, 9–23). However, despite some notable exceptions (e.g., (24–27)) most studies tend to focus on single species, single anesthetic, and hand-picked features of neural activity—such as spectral power or temporal entropy—potentially missing other important aspects of neural dynamics. Yet, the modern time-series literature is rich and vast; state-of-the-art extraction methods can span thousands of dynamical features that characterize time-series dynamics more exhaustively, from autocorrelation and statistics of the distribution, to signal complexity and self-similarity (28–30). How anaesthetics change the entire repertoire of brain dynamics across species, across spatial scales, and across pharmacological agents remains an open question.

**Figure 1.**
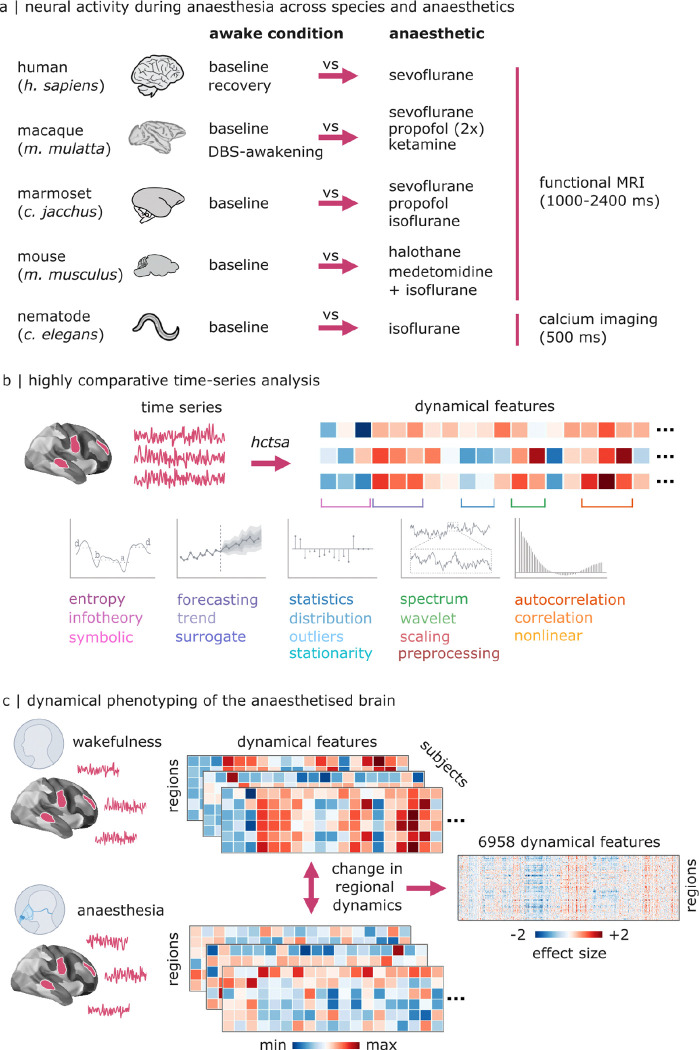
Systematic phenotyping of brain dynamics under anaesthesia (**a**) Overview of datasets and contrasts included in the present study. Human: awake vs vol 3% sevoflurane, and recovery vs vol 3% sevoflurane (31). Macaque (two datasets): awake vs sevoflurane; awake vs propofol; awake vs ketamine (15); and awake vs propofol (no DBS); central thalamus (CT) DBS versus propofol (no DBS); and CT DBS vs ventral thalamus (VT) DBS (32) Marmoset: awake vs sevoflurane; awake vs propofol; and awake vs isoflurane (32). Mouse: awake vs halothane; and awake vs medetomidine-isoflurane (14). Nematode: 0% isoflurane vs 4% isoflurane (33). For the mammalian species, time-series were acquired using functional MRI. For the nematode, time-series were acquired from GCamP calcium imaging. (**b**) Using *highly comparative time-series analysis* (hctsa (28)), we extract >6 000 dynamical features to characterise each time-series of regional brain activity. (**c**) Analytic strategy: for each brain region, we compare dynamical features during wakefulness and during anaesthesia. We obtain a regions-by-features matrix of effect sizes for each contrast, which can be compared across datasets. See Table S1 for full details on sample sizes, imaging techniques, and acquisition parameters description.

Here we systematically map how anaesthetics perturb the entire dynamic profile of the brain. We first compile a unique multi-scale dataset spanning invertebrate, murine and primate species undergoing imaging during loss and recovery of responsiveness induced by multiple volatile and intravenous anaesthetics, as well as reawakening from anaesthesia induced by deep-brain stimulation (DBS) of the macaque central thalamus (Fig. 1a). Focusing on effects that are shared across multiple anaesthetics enables us to exclude any physiological confounds that are specific to any one drug, and instead focus on what they all have in common: breakdown of responsiveness to the environment. We then apply massive temporal feature extraction to generate more than 6 000 univariate dynamical features that comprehensively characterise the neural activity of every cortical region/neuron (Fig. 1b). Comparing dynamical features across wakefulness and anaesthesia ((Fig. 1c)), we identify a phylogenetically conserved dynamical profile of anaesthetic-induced loss of consciousness and its restoration.

## RESULTS

Here we perform systematic phenotyping of the changes in local neural dynamics induced by different anaesthetic agents, across over 6 000 dynamical features of univariate time-series (28–30). We compile multiple datasets, each including at least one awake and one anaesthetised condition (Fig. 1a). Please see Methods for specific details regarding acquisition and processing of each dataset; an overview of the key parameters is provided in Table S1. Specifically, we include: a dataset of resting-state functional MRI data obtained from N = 15 healthy humans (*Homo sapiens*) who were scanned at baseline and during deep anaesthesia with the inhalational anaesthetic sevoflurane, as well as during spontaneous recovery of consciousness (31); a dataset of N = 5 macaque monkeys (*Macaca mulatta*) scanned several times during wakefulness and during anaesthesia with sevoflurane, propofol, or ketamine (15); a dataset of N = 4 marmoset monkeys (*Callithrix jacchus*) scanned during wakefulness and during anaesthesia with isoflurane, sevoflurane, or propofol (34); a dataset of N = 43 mice (*Mus musculus*) scanned either during wakefulness (N = 10) or during anaesthesia with halothane (N=19) or combined medetomidine-isoflurane (N = 14) (14). Since the effects of anaesthesia are shared even with invertebrates (6–8), we also include a dataset of N = 10 nematode worms (*Caenorhabditis elegans*) with calcium imaging acquired with or without isoflurane anaesthesia, having a comparable temporal resolution to our fMRI recordings (500ms) (35). For all the above datasets, we contrast every wakeful condition against every anaesthetised condition. Finally, we also include an additional macaque dataset, comprising N = 2 macaque monkeys scanned several times with fMRI during wakefulness, during propofol anaesthesia, and during anaesthesia combined with deep-brain stimulation of different thalamic nuclei: centromedian thalamus (CT), which induced awakening despite continuous anaesthetic infusion; and ventral-lateral thalamus (VT) which did not induce awakening (32). For this dataset, we contrast baseline wakefulness against propofol anaesthesia. We also contrast awakening induced by CT DBS during anaesthesia (i.e., propofol is present but the animal is awake), against anaesthesia without DBS, and against anaesthesia with VT DBS (which does not induce awakening). Inclusion of this unique dataset enables us to dissociate the presence of a high dose of propofol from its effect on wakefulness.

Starting from the preprocessed time-series of each brain region’s fMRI activity (respectively, each neuron’s calcium imaging activity in the nematode data) we extract its full dynamical profile across the most comprehensive available set of scientific time-series features, using the highly comparative time-series analysis (hctsa) toolbox (28, 29) (Fig. 1b). The hctsa features include methods from across the interdisciplinary scientific literature on time-series analysis, including biology but also physics, engineering, and economics. Features range from basic statistics of the distribution of time-points, outliers, periodicity, stationarity, to predictability, autocorrelation, self-affine scaling, and many more (28, 29). Each time-series feature takes a univariate time-series as input, and returns a single, real-valued summary statistic. Filtering for features that could not be computed for all regions/neurons yielded 6 958 features for further analysis.

For each dataset (e.g., marmoset, human), and for each wakefulness-anaesthesia contrast (e.g., baseline vs isoflurane anaesthesia; recovery versus sevoflurane; deep-brain stimulation vs no stimulation), we obtained the effect size of the statistical comparison of each timeseries feature in each region (Fig. 1c). Being expressed in units of standard deviation, effect sizes are comparable across features and also across regions and datasets. We measured effect size using Hedge’s *g*, which is interpreted in the same way as Cohen’s *d*, but more appropriate for small sample sizes (36). For each contrast, this procedure returned a matrix of size brain regions (neurons for the nematode) × 6 958 features.

### Systematic phenotyping of regional neural dynamics under anesthesia

**Figure 2.**
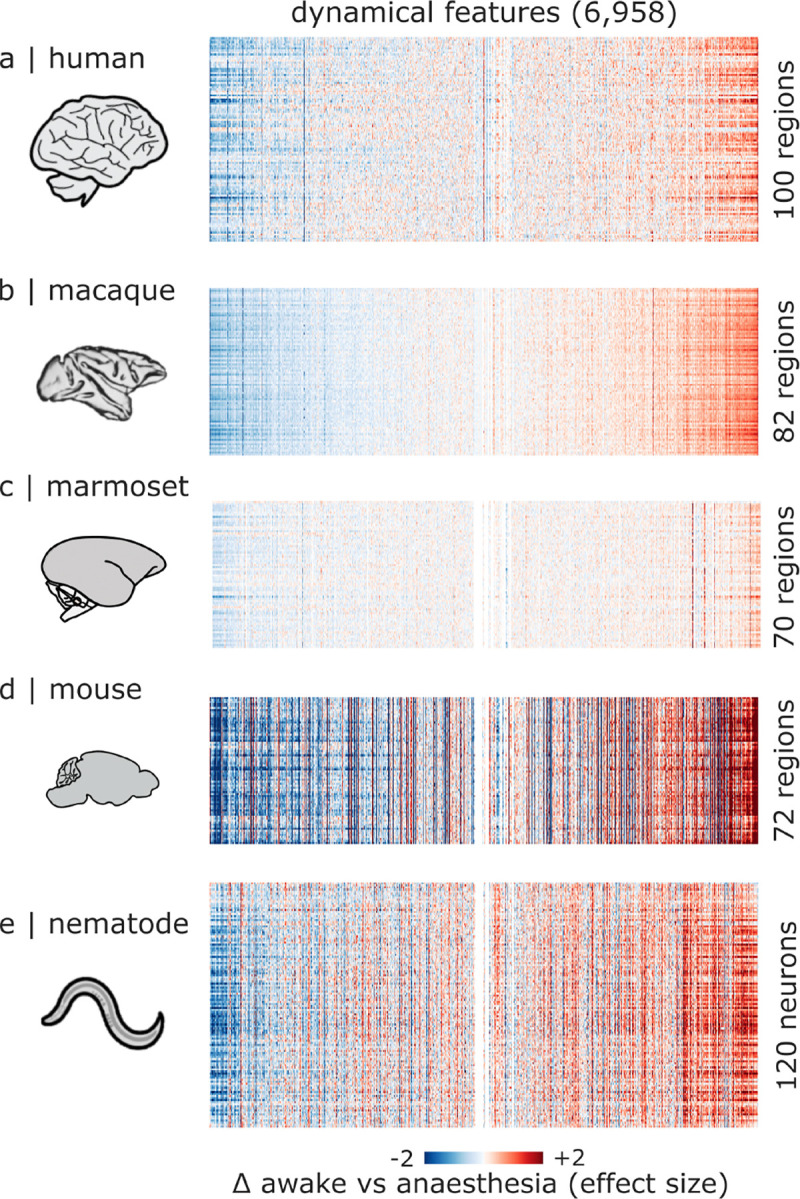
Anaesthetic-induced changes in neural dynamics across species (**a**) Human: mean effect sizes across awake vs vol 3% sevoflurane, and recovery vs vol 3% sevoflurane. (**b**) Macaque: mean effect sizes across awake vs sevoflurane; awake vs propofol; awake vs ketamine (for the Multi-anaesthesia dataset); awake vs propofol (no DBS); CT DBS versus propofol; and CT DBS vs VT DBS. (**c**) Marmoset: mean effect sizes across awake vs sevoflurane; awake vs propofol; and awake vs isoflurane. (**d**) Mouse: mean effect sizes across awake vs halothane; and awake vs medetomidine-isoflurane. (**d**) Nematode: effect size for awake vs isoflurane. Positive effect sizes indicate anaesthesia > no anaesthesia. The order of features (columns) is the same in each species, sorted to highlight common patterns. For visualisation purposes, the color range is capped at [−2, 2]. Fig. S1 shows the same data after column-wise z-scoring and reordering the rows, to highlight patterns of regional heterogeneity.

Fig. 2 shows the matrix of regions × dynamical features obtained by averaging across all contrasts and datasets for each species. Each regions-by-features matrix shows the effect size when contrasting the awake condition versus the anaesthesia condition, such that positive values indicate an increase during anaesthesia. As expected, there are some differences among the datasets, with the mouse and nematode data displaying greater sensitivity (evidenced by overall greater effect sizes), which may potentially be explained by the high spatial and temporal resolution of their respective acquisitions; namely, the mouse data has the fastest fMRI sampling and longest time-series, while the nematode data uses calcium imaging to measure activity of individual neurons at sub-second resolution (Table S1). Importantly, despite differences in acquisition and spatial resolution, processing strategies, model organism and anesthetic agent, we observe prominently aligned vertical bands among datasets, suggesting that diverse anesthetics elicit similar increases and decreases in local dynamics across species (Fig. 2).

**Figure 3.**
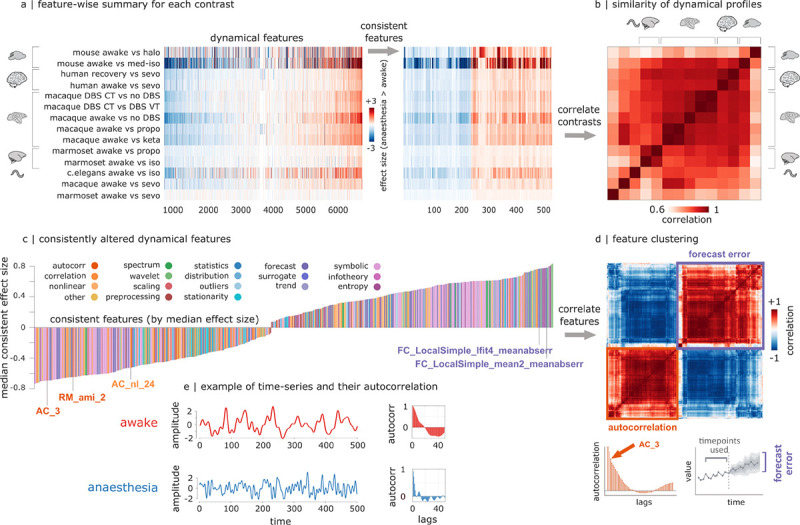
Consistent effects of anaesthesia on neural dynamics (**a**) Summary of feature-wise dynamical changes for each contrast and each species. For each contrast, effect sizes are averaged across brain regions/neurons to obtain a single value for each feature, representing its overall change across the brain. To identify dynamical features that are consistently altered across species and across anaesthetics, features are filtered to retain only those whose direction of effect is the same in each species (all positive or all negative). The outcome of this procedure is a set of 541 dynamical features that are consistently altered across regions/neurons, across anaesthetics, and across species. For visualisation purposes, the color range is capped at [−3, +3]. (**b**) The dynamical signatures of anaesthesia are positively correlated across species and across anaesthetics. (**c**) The median effect sizes are shown for each of the 541 consistent features, color-coded according to membership of 18 broad categories of dynamics (feature types). Exemplary features are highlighted, and illustrations are provided in the insets. (**d**) The consistent features are grouped into two main clusters, broadly pertaining to autocorrelation and forecasting error. (**e**) Examples of time-series from an awake (red) and an anaesthetised (blue) macaque, showing faster decay of autocorrelation under anaesthesia.

We next consider the phylogenetically-conserved dynamic profile associated with anesthesia. To identify time-series features that are consistently altered across species and across anaesthetics, we first average the feature-wise effect sizes across all regions and all awake-anaesthesia contrasts belonging to the same species. For each species, this generates one summary effect size per feature (Fig. 3a). Finally, features are filtered to retain only those whose direction of effect is the same in each individual contrast (all positive or all negative). we find 541 time-series features that are consistently altered across regions/neurons, across anaesthetics, and across species (Fig. 3a). This is significantly greater than expected by chance (*p* < 0.001), as assessed by a null model of 10,000 surrogate matrices where each contrast-feature pair is independently assigned a random sign (+1 or −1). Indeed, the probability of an individual feature exhibiting the same sign across 14 contrasts just by chance is 0.00012. We also find that the dynamical signatures of anaesthesia are highly correlated across different anaesthetics and different species (Fig. 3b; all *r* > 0.59, and all significant after FDR correction).

Broadly, we find two large clusters of time-series features that are consistently affected by anaesthesia (Fig. 3c,d). On one hand, anaesthesia reduces the value of features related to linear and nonlinear temporal autocorrelation in the brain (Fig. 3c,d). For example, some of the features exhibiting large reductions are *AC_3* and *RM_AMI_2*, which index the autocorrelation at lag 3 and automutual information at lag 2 of the timeseries, respectively. On the other hand, anaesthesia increases the error of forecasting the future of a time-series based on its past (Fig. 3c,d). For example, *FC_LocalSimple_lfit4_meanabserr* quantifies the mean absolute error of a linear fit from the past 4 time-points, and *FC_LocalSimple_mean2_meanabserr* reflects the mean absolute error of forecasting based on the mean of the last 2 time-points. Fig. 3e illustrates how these features capture changes in the underlying dynamics of neural time-series, focusing on autocorrelation as a feature that is straightforward to compute and readily interpretable. Additional examples for each contrast and dataset are provided in Fig. S2, and statistical comparisons for each contrast are provided for *AC_3* in Fig. S3 and for *RM_AMI_2* in Fig. S4. Altogether, we find that anaesthesia induces a breakdown in the relationship between past and future of neural activity.

**Figure 4.**
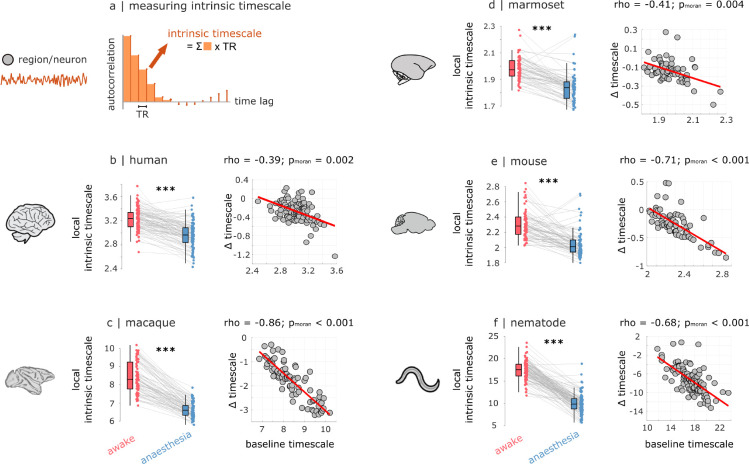
Anaesthesia reduces intrinsic neural timescales across species (**a**) Quantifying intrinsic neural timescales from spontaneous activity. For each region/neuron, the autocorrelation function is obtained by correlating the original time-series with a time-lagged version of itself, for increasing lags. Intrinsic neural timescale is computed by summing the values of the autocorrelation function (each multiplied by the TR) up to the point where autocorrelation drops to zero. A larger value of this measure therefore indicates a longer intrinsic timescale (41). (**b-f**) For each species (human, macaque, marmoset, mouse, and nematode), we obtain the mean intrinsic timescale of each region across wakefulness (red), and across anaesthesia conditions (blue). Within each species, we compare these distributions of regional intrinsic timescales. Each data-point represents one region/neuron. Box-plots: center line, median; box limits, upper and lower quartiles; whiskers, 1.5× interquartile range. **, *p* < 0.01; ***, *p* < 0.001, from non-parametric paired-samples t-test. We also show the correlation between anaesthetic-induced change in regional intrinsic timescale, and each region’s intrinsic neural timescale while awake. Significance of correlations is assessed against null models generated with Moran spectral randomisation (see Methods).

Temporal autocorrelation of spontaneous neural activity reflects how long neural information persists in a local brain area to influence its future behaviour: the “temporal receptive window” over which inputs coming into that brain region are integrated (2, 4, 37–40). Our data-driven analysis suggests that this fundamental organisational property of the brain is compromised under anaesthesia. We therefore explicitly quantify the intrinsic timescale at which each region operates, defined as in (41) as the product between repetition time (TR) and the sum of autocorrelation function values up to the point where autocorrelation drops to zero, such that the future of the time-series becomes independent of its past (Fig. 4a). A larger value of this measure indicates a longer intrinsic timescale (41). For each species (human, macaque, marmoset, mouse, and nematode), we compare the distribution of regional intrinsic timescales between wakefulness and anaesthesia. Within every single species we find that on average across regions, intrinsic neural timescales are significantly reduced under anaesthesia (Fig. 4b–f; results at the level of each contrast are provided in Fig. S5). This effect is neither uniform across regions, nor random. Rather, anaesthetic-induced reduction of intrinsic timescales is proportional to each region’s baseline level of intrinsic timescale, such that regions/neurons with longer intrinsic timescales see the most severe reductions, indicated by significant negative correlations across regions between baseline level and anaesthetic-induced change (Fig. 4b–f). Altogether, at the temporal resolution afforded by fMRI we find that anaesthesia consistently induces a compression of the brain’s intrinsic timescales, leading to reduced local persistence of information, and shorter temporal windows for integration.

### Mapping local neural dynamics to gene expression across phylogeny

**Figure 5.**
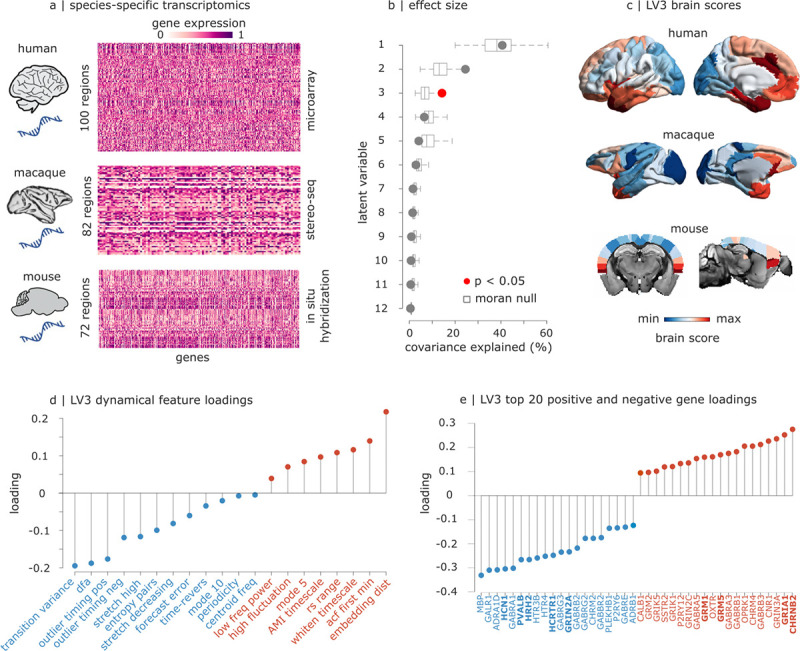
Anaesthetic-induced changes in neural dynamics map onto phylogenetically conserved dimensions of cortical gene expression (**a**) We obtain species-specific cortical expression of key brain-related genes from microarray (human), stereo-seq (macaque) and in-situ hybridization (mouse). Experssion of each gene is sigmoid-normalised to lie in the interval between 0 and 1. (**b**) We find a statistically significant latent dimension of multivariate association between gene expression and anaesthetic-induced feature change (LV3). The first two latent dimensions are not significant beyond the effect of spatial autocorrelation.(**c**) Representation of the significant LV3 on the cortex of each species, delineating a phylogenetically conserved anterior-posterior gradient.(**d**) Loading of each representative dynamical feature onto the significant dimension of multivariate association with gene expression. (**e**) Top 20 genes in either direction whose regional expression drives the association with anaesthetic-induced changes across species. Red indicates positive loading, blue indicates negative loading. See Fig. S6 for the full set of gene loadings.

So far, we observed that diverse anaesthetics induce consistent changes in neural dynamics across species. Since anaesthetics perturb local biophysics by engaging a range of receptor systems and triggering molecular cascades, we next ask whether these phylogenetically conserved changes in local dynamics might be underpinned by phylogenetically conserved patterns of gene expression. To address this question, we use comprehensive species-specific databases of cortical transcriptomics for human (microarray (42)), macaque (stereo-seq (43)), and mouse (in situ hybridization (44)) (Fig. 5a). For each species, we map spatial patterns of cortical expression for a previously-assembled list of brain-related genes (45). This list includes genes pertaining to neurotransmitter and neuropeptide receptors, myelin, and interneuron cell-type markers (parvalbumin, somatostatin, calbindin, vasoactive intestinal polypeptide) (45, 46). After accounting for all preprocessing criteria, such as intensity filtering, the final set comprises 81 orthologous genes that are available in each of our three transcriptomic databases (see Table S3 and Table S4 for the list of gene names).

We then use partial least squares correlation (PLS) to integrate cortical transcriptomics with dynamical features (each concatenated across species, after z-scoring within species). As a form of unbiased feature selection to aid interpretation, we use a reduced set of 20 time-series features that have been shown to be sensitive to a wide range of dynamical phenomena in naturally occurring and artificial systems (47). This data-driven set (known as the catch22 set (47)) includes features pertaining to autocorrelation, forecasting, and temporal frequency, capturing the dynamical properties that we found to be consistently perturbed by anaesthesia. From the original 22 features, we exclude one feature that did not meet all our filtering criteria in all species; a second feature occasionally produced no variance for individual regions and was therefore also excluded from the regionally-specific PLS analysis, leaving 20 features; see Methods and Table S2). PLS is a data-driven technique that identifies multivariate patterns of maximum covariance between datasets—in this case, gene expression and changes in dynamical features, each concatenated across species (see Methods) (48, 49). We assess significance against a distribution of autocorrelation-preserving null models generated using Moran spectral randomisation for each species (see Methods), embodying the null hypothesis that local dynamics and gene expression are spatially correlated with each other purely because of inherent spatial autocorrelation (50, 51).

We find a statistically significant latent variable (linear weighted combination of the original variables) relating anaesthetic-induced changes in local dynamics, and cortical patterns of gene expression, across species. This latent variable (LV3) explains significantly more covariance than explained by spatial autocorrelation alone (17%; by contrast, LV1 and LV2 are not significant, after taking into account spatial autocorrelation). This significant latent variable is associated with consistent cortical patterns in each species, which can be broadly characterised as anterior-to-posterior (Fig. 5c). At one end, LV3 relates measures of outliers and forecasting error with the expression of a marker gene for inhibitory interneurons (*PVALB*), as well as histamine receptor (*HRH2*) and orexin/hypocretin receptor (*HCRTR1*), the pace-maker channel *HCN1*, and NMDA glutamate receptors (Fig. 5d,e). At the other end, LV3 relates changes in measures of autocorrelation with muscarinic and nicotinic cholinergic receptors (*CHRNB2*, *CHRM4*), as well as with metabotropic (*GRM5*) and ionotropic AMPA (*GRIA1*) glutamate receptors (Fig. 5d,e). Altogether, we find that phylogenetically conserved anaesthetic-induced changes in local dynamics are subtended by a phylogenetically conserved anterior-posterior transcriptomic gradient, encompassing genes pertaining to regulation of arousal and sleep-wake regulation—possibly indicating shared neurobiological circuitry on which anaesthesia operates.

### From local dynamics to global networks

**Figure 6.**
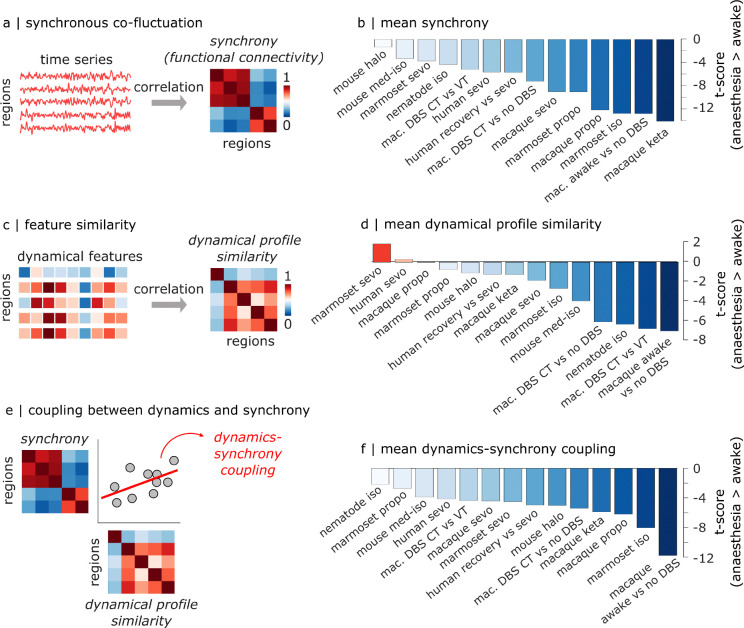
Anaesthesia decouples temporal and feature-based representations of inter-regional interactions (**a**) Synchrony is measured as the magnitude of correlation between two regions’ time-series over time, reflecting co-fluctuation. (**b**) Across species and anaesthetics, anaesthesia significantly reduces the mean magnitude of synchronous co-fluctuations of neural activity. (**c**) Dynamical profile similarity (DPS) is measured as the magnitude of correlation between the dynamical features of two regions’ time-series. For both synchrony and DPS, the result is a region-by-region matrix of pairwise similarities. (**d**) Anaesthesia significantly reduces the mean similarity between regions’ dynamical profiles. (**e**) Coupling between synchrony and dynamics is obtained by correlating the matrix of inter-regional synchrony and the matrix of inter-regional dynamical profile similarity. It quantifies the extent to which regions with similar temporal co-fluctuations also exhibit similar time-series features. (**f**) Across species and anaesthetics, anaesthesia reduces the coupling between synchrony and dynamics.

Up to this point we have focused on local dynamics and identified their local neurobiological signatures. However, individual neurons and regions are embedded in a larger synaptic network. In a complex network such as the brain, changes in regional dynamics may both shape and be shaped by changes in inter-regional functional interactions. For instance, if two populations begin to operate at faster timescales, what effect does this have on their capacity to spontaneously synchronize with each other? To investigate the relationship between local dynamics and network-wide communication, we estimate two measures of inter-regional dependence (Fig. 6a). First, we compute the temporal synchrony of co-fluctuations in neural activity, operationalised as the magnitude of functional connectivity (zero-lag Pearson’s correlation between BOLD time-series; Fig. 6a). Second, we compute the magnitude of pairwise similarity between regional dynamical features generated by our large set of time-series features (30), measuring the dynamical profile similarity (DPS) of two neuronal populations (30)(Fig. 6c). The two measurements allow us to identify distributed regions that potentially display common local dynamics—suggesting common function—without necessarily displaying time-locked activity.

Across species and anaesthetics, anaesthesia does not only change the dynamical properties of individual regions: it also weakens the relationships between the dynamical profiles of different regions, significantly reducing their similarity (Fig. 6d). At the same time, we find that anaesthesia desynchronises the interactions between different regions, across all species and anaesthetics (Fig. 6b). Additionally, in the awake brain, regions (or neurons) with similar univariate dynamics—suggesting similar computational properties—tend to display time-locked activity, indicating that they also perform the same function (Fig. 6e). However, the coupling between synchrony and dynamics is ubiquitously compromised under anaesthesia (Fig. 6f; see also Fig. S7 for statistical comparisons for each contrast and dataset). In other words, in the anaesthetised brain, regions (or neurons) with similar univariate dynamics are less likely to exhibit coordinated fluctuations over time. Given this relationship between connectivity and local dynamics, we next turn our attention to how the effect of anaesthesia on circuit dynamics can be modeled and tuned.

### Computational modelling of anaesthetised neural dynamics

**Figure 7.**
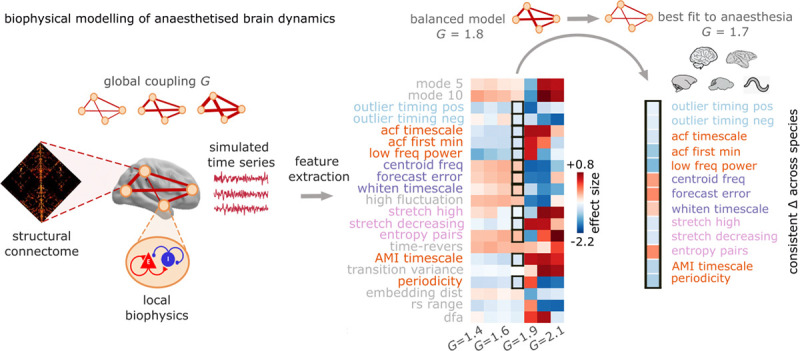
Computational modelling reveals that anaesthetic-induced changes in neural dynamics are consistent with weakened inter-regional structural coupling (**a**) The dynamic mean-field model comprises two key ingredients: a model of regional local dynamics (here, coupled excitatory and inhibitory neural masses) and a wiring diagram of inter-regional coupling (here, the empirical human structural connectome, reconstructed from diffusion tractography). The model has one free parameter, the global coupling *G*, which scales the inter-regional connectivity. To obtain an independently parametrised model, *G* is set to the value that best matches the empirical fMRI data (here, *G* = 1.8; see Fig. S8). (**b**) The model is used to simulate regional BOLD time-series for values of *G* from 1.4 to 2.1, in 0.1 increments (N = 40 simulations per value). For each simulated time-series, we extract 21 regional dynamical features from the catch22 representative subset of hctsa features, and compare them against the features obtained from the tuned model. 13 of the catch22 features are consistently affected by anaesthesia across species, and we find that each of them exhibits a change in the same direction as anaesthesia when the model is set to *G* = 1.7, corresponding to weaker global structural coupling. At *G* = 1.7, the model also exhibits diminished coupling between synchrony and DPS, as observed under anaesthesia (Fig. S9).

Finally, having identified a consistent dynamical signature of anaesthesia across species and across anaesthetics, we seek to understand its mechanistic origin through computational modelling. Network-based computational models provide a way to link specific perturbations to their functional consequences, by simulating BOLD time-series (52–56). Specifically, we use a computational neural mass model of excitatory and inhibitory populations, coupled according to the empirical structural connectivity of the human brain (57–59) (Fig. 7). The model has one free parameter, the global coupling *G*, which controls the overall strength of signal transmission between brain regions by uniformly scaling the value of every weight in the structural connectome. The resulting scaled connectome represents the effective connectivity between regions that is used to generate the simulated brain dynamics. We first tune *G* to match an independent high-quality dataset of fMRI signals from healthy humans, from the Human Connectome Project (see Methods). Then, starting from this independently parameterised model (obtained for *G* = 1.8; Fig. S8), we systematically vary the *G* parameter from 1.4 to 2.1, in increments of 0.1 (Fig. 7). At each value of *G* we simulate regional BOLD signals for a sample of *N* = 40 simulations. For each region in each simulation at each value of *G*, we then extract the catch22 reduced set of dynamical features that have been shown to be representative of the broader literature on dynamical systems (47) (as above, one of the 22 features in catch22 features was not included because it failed our initial filtering step, as described in the Methods; a second feature that had to be excluded from the PLS analysis due to some regions with no variance, was included in the next analysis by ignoring non-finite values were ignored when computing the brain-wide mean value; hence, 21 features were included in this analysis, shown in Table S2). Out of the resulting 21 dynamical features, 13 exhibit consistent changes across species as a result of anaesthesia, providing a lower-dimensional dynamical phenotype of anaesthesia that we can try to match with our model (Fig. 7).

We ask: can we reproduce the same pattern of changes in dynamical features by modulating the global coupling? We find that this is the case upon reducing the global coupling to *G* = 1.7, where compared against the tuned model (*G* = 1.8), every one of our 13 features of interest exhibits a change in the same direction as what is observed with anaesthesia across species (Fig. 7). Additionally, we also see that a reduction in the global effective coupling *G* induces a reduction of the association between synchrony and dynamical profile similarity (Fig. S9), again consistent with our empirical observations across species and anaesthetics. Note that synchrony-dynamics coupling was not part of the dynamical phenotype that our model was explicitly optimised to match. Rather, a biologically plausible change in the global coupling between synchrony and dynamics arises spontaneously, when the model is optimised to match the effect of anaesthesia on a distinct set of local dynamical features. Altogether, our phenomenological computational model reveals that the signatures of anaesthesia in the regional dynamics and inter-regional interactions of the brain (lower autocorrelation, increased forecasting error, and weaker association between synchrony and dynamics) could be explained by a mechanism of reduced effective coupling between regions. In other words, it is possible to reproduce the neural effects of anaesthesia by reducing regions’ capacity to transmit signals and interact with each other via white matter connections.

### Sensitivity and validation

To ensure that results are not due to specifics of data selection, processing and analytic choices, we conduct additional sensitivity analyses and validation. We show that the PLS models for the association between anaesthetic-induced changes in local neural dynamics and cortical transcriptomics remain the same when the analysis is repeated using all dynamical features that are consistently perturbed by anaesthesia across species, instead of the reduced set from catch22 (Fig. S10). We observe the same significant LV3, with the same anterior-posterior cortical gradient across species, alongside correlated gene loadings (Fig. S11). Results of this analysis also remain robust upon including additional contrasts for the human sevoflurane dataset, namely 2% vol and approximately 4.4% vol, i.e., both lower and higher than the dose used for our main analysis (Fig. S12). Likewise, results from the analysis with all consistent features remain robust upon excluding the nematode data in the analysis, showing that inclusion of this non-mammalian, non-fMRI dataset does not bias the findings (Fig. S13).

To provide further validation, we show that the PLS brain scores obtained from human microarray transcriptomics are strongly and significantly correlated with the brain score obtained from human RNA-seq gene expression data instead (Fig. S14). Likewise, the PLS brain scores obtained from the *in situ hybridization* mouse database can be reproduced when using mouse gene expression from two recently released alternative databases using MERFISH (60, 61) (Fig. S15). Additionally, one of the genes exhibiting the strongest spatial association with the significant latent variable is *PVALB/Pvalb*, which encodes the protein parvalbumin, a marker of inhibitory interneurons. We validate this finding in the macaque brain, for which immunohistochemically-derived parvalbumin density data are available for several cortical regions (62). As expected, we find a significant negative spatial correlation between the PLS brain scores for macaque, and the regional protein density of parvalbumin, consistent with the negative loading of the *PVALB* gene (Fig. S16). Thus, we show that our gene-dynamics association is robust both to use of a broader set of dynamical features, and to use of a different modality for human and mouse gene expression.

## DISCUSSION

Processing information to guide engagement with the environment is a fundamental function of all nervous systems. Anaesthetics modulate neural dynamics to temporarily shut down this fundamental function, consistently suppressing sensation and action (6). Being consistently observed across species, this phenomenon hints at the existence of phylogenetically conserved circuit dynamics for information processing, which would then be targeted by anaesthesia. Here we systematically sampled over 6 000 features of local neuronal dynamics, searching for a conserved dynamical signature of anaesthesia across imaging modalities, species, and anaesthetics. We identified an evolutionarily conserved dynamical profile of anaesthesia. This suggests that it is not just the behavioural response to anaesthesia (isolation from the environment) that is common across species and anaesthetics, but also individual features of neural activity. A shared dynamical signature of anaesthesia dovetails with a broader appreciation for not only the contribution of structure (10, 11, 14, 15, 22, 63), but also its emergent dynamics (5, 9, 12, 13, 16–21, 23–25, 64, 65).

The dynamical profile of anaesthesia is enriched for multiple time-series features related to the temporal predictability of the signal’s future from its past, which we find to be consistently reduced, coinciding with lower autocorrelation. Although they originate from different fields of science and perform different computations on the input neural signals, these features broadly measure the relationship between past and future of a time-series. Examples include features that measure recurring motifs in time-series, outlier features that identify unexpected patterns, features that measure the error of forecasting the signal’s future based on its past, and linear and non-linear measures of autocorrelation. This is conceptually related to the notion of intrinsic neural timescale: in the awake mammalian brain, short timescales are thought to encode rapidly-changing sensory information. Conversely, long timescales reflect longer temporal receptive windows, supporting integrative processes and the encoding of contextual information (2, 4, 37–41, 66–71). Our findings suggest that in the anaesthetised brain, intrinsic timescales become systematically shorter. In turn, shorter time-scale means that the brain is less capable of encoding contextual information in its ongoing dynamics, and less capable of integrating information over time (4, 68, 69, 72). This faster decay of local activity may explain why under anaesthesia incoming stimuli fail to escalate the processing hierarchy and achieve global relevance (7, 32, 73–75), up to the point that the organism will fail to respond even to noxious stimuli. Indeed, we also observed weaker synchrony between different regions under anaesthesia, which could be reproduced in our computational model by reducing the effectiveness of connectome-mediated signal tranmission between regions. Collectively, these dynamical features suggest that under anaesthesia, brain regions become functionally isolated in both time and space: each region is less able to influence other regions (reduced effective coupling, and reduced functional synchrony) and even less able to influence its own future activity, as indicated by weaker past-future relationships in the signal.

At first blush, the shorter intrinsic timescale in the BOLD signal during anaesthesia may appear at odds with results from electrophysiology. Anaesthesia is often associated with slowing down of cortical electrodynamics, as measured by increased prevalence of electrophysiological slow-waves, slower response time to perturbations in macaque local field potentials (LFPs), and longer autocorrelation of the EEG signal (4, 21, 76–80). However, both the haemodynamics and calcium imaging used here measure neural activity at several orders of magnitude slower than EEG and LFPs (ranging from 500ms for our GCamP data in *c.elegans* to 2400ms for the fMRI, as opposed to the millisecond-level resolution of electrophysiology). Shorter fMRI timescale is therefore not inconsistent with slowing down of EEG activity. To provide practical support for this theoretical argument, we also provide a toy example, showing that the same time-series can appear slower and more synchronised or faster and less synchronised, depending on whether they are sampled at a fast or slow rate.

Indeed, shorter fMRI intrinsic timescale has been reported in humans under deep general anaesthesia (but not under mere sedation) and in patients with disorders of consciousness (81); reduced fMRI autocorrelation was also reported in anaesthetised macaques (11). Likewise, under anaesthesia, human fMRI exhibits a reduction in the power of slow frequencies (82); a shift to faster neuronal activity was also reported in anaesthetised *c.elegans* with 2Hz calcium imaging (35), and medetomidine-isoflurane anaesthesia in mice is known to shift the spectral components of fMRI signal fluctuations towards higher frequencies (83, 84)). Together with the findings in the present report, these observations in fMRI and electrophysiology collectively suggest convergence towards a narrower, less diverse range of timescales during anaesthesia, with infraslow fMRI becoming faster, and fast electrophysiology becoming slower.

If the effects of anaesthesia on local dynamics are stereotyped across species, do they also have a common molecular origin? We find that the effect of anaesthesia on local neural dynamics co-varies spatially with an anterior-posterior transcriptomic gradient in the mammalian cortex. This anterior-posterior gradient encompasses genes pertaining to inhibitory interneurons (*PVALB*) as well as regulation of arousal and sleep-wake cycles (cholinergic receptors, orexin/hypocretin, histamine), many of which are thought to be involved in the causal mechanisms of sleep and its pharmacological counterpart, anaesthesia (6, 9, 20, 78, 85–90). For example, orexin/hypocretin neurons control the stability of sleep/wake dynamics (89), and loss of these neurons causes the sleep disorder narcolepsy in humans and other mammals (91–93). Notably, hyperpolarization-activated cyclic-nucleotide (HCN) gated channels, which are present both in mammalians and invertebrates and play a pace-maker role in the brain (94), are sensitive to many volatile and intravenous anaesthetics (6, 95–97). Indeed, *HCN1* inhibition was recently identified as a shared mechanism of propofol and ketamine anaesthesia in humans (20). Altogether, we find that the phylogenetically conserved changes in local neuronal dynamics induced by anaesthesia are underpinned by corresponding phylogenetically conserved patterns of gene expression. These results lend support to the growing appreciation for the role of genetic and molecular contributors to neural dynamics (1, 2, 9, 30, 98–101).

Perturbations of local dynamics also manifest as changes in inter-regional communication. In addition to reshaping local dynamics, we find that anaesthesia desynchronises regions with similar dynamical profiles, such that they no longer engage in time-locked activity even though they have similar ongoing dynamics. Corroborating the present results, reduced BOLD signal co-fluctuations were previously identified with a variety of anaesthetics in humans (81, 102–104), macaques (11), marmosets (105), and rats (106), but decorrelations have also been reported at the neuronal level in flies and mice (107, 108). Here we confirm that this desynchronisation of slow neural dynamics generalises across scales, from neuronal calcium imaging and mammalian haemodynamics. Notably, Huang and colleagues reported lower synchrony and reduced autocorrelation as shared fMRI signatures of unconsciousness between anaesthesia and disorders of consciousness, hinting at an even more fundamental link with consciousness (81). Indeed, spontaneous cognition does not happen in isolation, but requires coordination between different macroscale functional circuits, manifesting as synchronous fluctuations—which are lost when consciousness is suppressed (72).

Notably, we also find that anaesthetic-induced desynchronisation has the effect of bringing out of step regions with similar dynamical phenotypes. Mechanistically, our connectome-based computational model indicates that the dynamical signatures of anaesthesia on synchrony and intrinsic timescale are both compatible with a reduction of the effective coupling between regions, reflecting impairment of connectome-mediated signal transmission (109). Although our model does not address the specific biological mechanisms that result in weaker signal transmission, this computational account of reduced inter-regional effective coupling at the macroscale is consistent with empirical reports at the microscale, showing that desflurane anaesthesia reduces the strength of excitatory synaptic connectivity between neurons of rat visual cortex (110)). Mechanistically, a failure of synaptic transmission would be consistent with the “synaptic power failure” account of anaesthesia, whereby volatile anaesthetics hinder excitatory synaptic function by decreasing the availability of presynaptic ATP (111, 112). More broadly, our finding that weaker effective connectivity leads to less temporal autocorrelation converges with recent modelling results, showing that timescales in autocorrelation increase with the effective strength of recurrent interactions in network models (113, 114). Thus, anaesthesia may reduce recurrent interactions in the brain, mainfesting as reduced intrinsic timescale.

Putting the region- and network-level perspectives together, we find that under anaesthesia regions become less coordinated with other regions at the same point in time (desynchronisation) and also less coordinated with their own future activity over time (faster decay of the autocorrelation and shorter intrinsic timescales). In other words, neural activity under anaesthesia remains more locally isolated, both spatially and temporally. These results are in excellent agreement with recordings from local field potentials and intracranial electrocorticograms in humans, which showed that under propofol anaesthesia, neuronal activity becomes desynchronised and functionally fragmented across both time and space (115). Anaesthetic-induced breakdown of the relationship between local and global activity is also consistent with work showing that under anaesthesia and sleep, both spontaneous and stimulus-evoked activity fails to propagate globally (32, 75, 116, 117). Our haemodynamic and calcium imaging findings therefore converge with these theoretical predictions and electrophysiological observations: under anaesthesia we observe more locally constrained neural activity, and a loss of coordination between regions whose dynamical phenotypes suggest shared computational properties.

Rich phenotyping of local dynamics provides deeper insight into the effects of additional experimental manipulation. For example, we find that dynamical signatures of anaesthesia are reversed upon reawakening from anaesthesia induced by centromedian thalamic deep-brain stimulation in the macaque (32). We demonstrate this bi-directional association by contrasting propofol anaesthesia with centromedian thalamic DBS, which restores arousal and responsiveness to the environment. We also included an even more stringent contrast between centromedian thalamic DBS, and DBS in a control site (ventrolateral thalamus), which fails to reawaken the animal from anaesthesia despite equivalent level of stimulation (32). Collectively, these experiments show that the dynamical signatures do not merely indicate the presence of anaesthetic agents in the system; rather, they reflect the suppression of information processing.

A multivariate dynamical signature of anaesthesia that is consistent across species also opens the possibility to reproduce anaesthetic effects with greater fidelity *in silico*. Existing computational models of BOLD signal changes under anaesthesia are typically tuned to reproduce a small number of experimentally observed features (often just one), such as static or dynamic functional connectivity, global synchrony, or metastability (109, 118–122). Here we show that it is possible to capture a richer readout of dynamical features. Note that our model is not intended to depict the accurate molecular neurobiology of any specific anaesthetic drug. Rather, by identifying a simple and intuitive mechanism that reproduces the empirical observations, this model could be used to make further, experimentally testable predictions about what other phenomena should arise from the same kind of mechanism, but have not yet been observed. When validated or refuted *in vivo*, such predictions can then guide the development of more refined models *in silico*, progressively converging on the underlying biological truth (56, 123). For example, future work could extend this modelling approach to systematically assess different pharmacological and stimulation protocols for reversing the effects of anaesthesia, and then validate the most promising ones in animal models. Ultimately, a combined *in silico* and *in vivo* approach for translational discovery may potentially be used to identify novel targets for treating disorders of consciousness. Indeed, as discussed in the previous paragraph, these dynamical signatures of anaesthesia can be selectively reversed by targeted deep-brain stimulation. Collectively, these empirical and computational results bring into focus the mapping between local dynamics and global interactions, and how they can be bi-directionally modulated by pharmacology and stimulation.

The consistency of dynamical effects across species suggests that dynamical phenotypes may provide a common frame of reference for phylogenetic comparison. Identifying mappings between the brains of humans and other species is a perennial question of translational neuroscience. The traditional approach has focused on anatomy, whereby homologous brain areas are identified according to shared morphological or cytoarchitectonic properties, or similar connectional patterns (124–129). More recently, gene expression has also been used as a high-dimensional common space to embed the cortical architecture of different species, such as human and mouse, without the need to explicitly match regions (45, 130, 131). A complementary approach involves finding homologs by identifying which brain regions exhibit consistent responses to the same stimuli (132–136), or matching recurrent patterns of whole-brain activity (5, 137, 138). Here, we combined these two approaches: we used the high-dimensional common space of local dynamics to enable inter-species comparison in response to the same intervention (anaesthesia). Since dynamics are ubiquitous across nervous systems, our findings show that dynamical phenotyping may provide a common reference frame to embed a wide and diverse range of neuroscientific datasets, from human functional MRI to nematode calcium imaging.

It is especially noteworthy that our results are observed across both regional haemodynamics (from BOLD fMRI and fMRI with monocrystalline iron oxide nanoparticle contrast agent) but also single-neuron data from calcium imaging. This means that any of our results that are shared across species cannot logically be attributed to putative physiological confounds of the haemodynamic signal such as breathing, cardiac, or vasodilation/vasoconstriction differences. First, because the different anaesthetics included here do not all influence such physiological parameters in the same way; for example, volatile anaesthetics are vasodilators, whereas propofol is a vasoconstrictor (6–8, 78, 139). The second reason is that our contrasts include reawakening induced by thalamic DBS during which a high dose of propofol is still present (32). Third, because such extraneous physiological effects are simply not present in the nematode worm, unlike the behavioural effects of anaesthesia, and unlike our results about neural dynamics, which are shared across species. Therefore, generalisation of our results to nematode calcium imaging represents a strong control that our results cannot be attributed to any fMRIspecific confounds.

The present work should be considered in light of multiple methodological limitations. For example, we did not have the same anaesthetic in every species, introducing heterogeneity of molecular and cellular mechanisms. However, our focus here was on anaesthesia’s effect on the brain’s capacity to process environmental information, which is indexed by loss of behavioural responsiveness—a marker that is widely shared across species and anaesthetics (6, 140). Combining datasets from different species and different modalities is also challenging due to methodological heterogeneity in acquisition modality and preprocessing. Such differences in anaesthetics and acquisition methodologies across datasets could represent a limitation, if the present study were focused on inter-species differences. However, our interest is rather in the consistencies across these species and anaesthetics. In this context, differences in how the various datasets were acquired and processed (e.g., with vs without global signal regression) may even be seen as an asset: convergence of results across extraneous methodological variations can be interpreted as an additional support for the robustness of our findings.

This being said, we acknowledge that inter-species differences are also a fundamental topic of investigation, which our present datasets are not well suited for addressing. Future work with standardised protocols for neuroimaging acquisition and anaesthetic administration will be required to fully answer this question. Altogether, through systematic dynamical phenotyping across species we have identified an evolutionarily conserved dynamical regime of impaired information processing, which is under bi-directional causal control by global pharmacological intervention and local stimulation.

## METHODS

### Human fMRI anaesthesia dataset

The human sevoflurane data included in this study have been published before (31, 141, 142). For clarity and consistency of reporting, we use the same wording as in those previous publications, and we refer the reader to the original publication for details (31).

#### Participants and ethics

The ethics committee of the medical school of the Technische Universitat Munchen (Munchen, Germany) approved the current study, which was conducted in accordance with the Declaration of Helsinki. Written informed consent was obtained from volunteers at least 48 h before the study session. Twenty healthy adult men (20 to 36 years of age; mean, 26 years) were recruited through campus notices and personal contact, and compensated for their participation in the study. Before inclusion in the study, detailed information was provided about the protocol and risks, and medical history was reviewed to assess any previous neurologic or psychiatric disorder. A focused physical examination was performed, and a resting electrocardiogram was recorded. Further exclusion criteria were the following: physical status other than American Society of Anesthesiologists physical status I, chronic intake of medication or drugs, hardness of hearing or deafness, absence of fluency in German, known or suspected disposition to malignant hyperthermia, acute hepatic porphyria, history of halothane hepatitis, obesity with a body mass index more than 30 kg/m2, gastrointestinal disorders with a disposition for gastroesophageal regurgitation, known or suspected difficult airway, and presence of metal implants. Data acquisition took place between June and December 2013.

#### Anaesthesia protocol

Sevoflurane concentrations were chosen so that participants tolerated artificial ventilation (reached at 2.0 vol%) and that burst-suppression (BS) was reached in all participants (around 4.4 vol%). To make group comparisons feasible, an intermediate concentration of 3.0 vol% was also used. In the MRI scanner, participants were in a resting state with eyes closed for 700s. Since EEG data were simultaneously acquired during MRI scanning (31) (though they are not analysed in the present study), visual online inspection of the EEG was used to verify that participants did not fall asleep during the pre-anaesthesia baseline scan. Sevoflurane mixed with oxygen was administered via a tight-fitting facemask using an fMRI-compatible anaesthesia machine (Fabius Tiro, Drager, Germany). Standard American Society of Anesthesiologists monitoring was performed: concentrations of sevoflurane, oxygen and carbon dioxide, were monitored using a cardiorespiratory monitor (DatexaS, General electric, USA). After administering an end-tidal sevoflurane concentration (etSev) of 0.4 vol% for 5 min, sevoflurane concentration was increased in a stepwise fashion by 0.2 vol% every 3 min until the participant became unconscious, as judged by the loss of responsiveness (LOR) to the repeatedly spoken command “squeeze my hand” two consecutive times. Sevoflurane concentration was then increased to reach an end-tidal concentration of approximately 3 vol%. When clinically indicated, ventilation was managed by the physician and a laryngeal mask suitable for fMRI (I-gel, Intersurgical, United Kingdom) was inserted. The fraction of inspired oxygen was then set at 0.8, and mechanical ventilation was adjusted to maintain end-tidal carbon dioxide at steady concentrations of 33 ± 1.71 mmHg during BS, 34 ± 1.12 mmHg during 3 vol%, and 33±1.49 mmHg during 2 vol% (throughout this article, mean ± SD). Norepinephrine was given by continuous infusion (0.1±0.01*μg* kg-1 min1) through an intravenous catheter in a vein on the dorsum of the hand, to maintain the mean arterial blood pressure close to baseline values (baseline, 96 ± 9.36 mmHg; BS, 88 ± 7.55 mmHg; 3 vol%, 88 ± 8.4 mmHg; 2 vol%, 89±9.37 mmHg; follow-up, 98±9.41 mmHg). After insertion of the laryngeal mask airway, sevoflurane concentration was gradually increased until the EEG showed burst-suppression with suppression periods of at least 1,000 ms and about 50% suppression of electrical activity (reached at 4.34 ± 0.22 vol%), which is characteristic of deep anaesthesia. At that point, another 700s of electroencephalogram and fMRI was recorded. Further 700s of data were acquired at steady end-tidal sevoflurane concentrations of 3 and 2 vol%, respectively (corresponding to Ramsay scale level 6, the deepest), each after an equilibration time of 15 min. In a final step, etSev was reduced to two times the concentration at LOR. However, most of the participants moved or did not tolerate the laryngeal mask any more under this condition: therefore, this stage was not included in the analysis. Sevoflurane administration was then terminated, and the scanner table was slid out of the MRI scanner to monitor post-anaesthetic recovery. The participants was manually ventilated until spontaneous ventilation returned. The laryngeal mask was removed as soon as the patient opened his mouth on command. The physician regularly asked the participant to squeeze their hand: recovery of responsiveness was noted to occur as soon as the command was followed. Fifteen minutes after the time of recovery of responsiveness, the Brice interview was administered to assess for awareness during sevoflurane exposure; the interview was repeated on the phone the next day. After a total of 45 min of recovery time, another resting-state combined fMRI-EEG scan was acquired (with eyes closed, as for the baseline scan). When participants were alert, oriented, cooperative, and physiologically stable, they were taken home by a family member or a friend appointed in advance.

#### MRI data acquisition

Although the original study acquired both functional MRI (fMRI) and electroencephalographic (EEG) data, in the present work we only considered the fMRI data. Data acquisition was carried out on a 3-Tesla magnetic resonance imaging scanner (Achieva Quasar Dual 3.0T 16CH, The Netherlands) with an eight-channel, phased-array head coil. The data were collected using a gradient echo planar imaging sequence (echo time = 30 ms, repetition time (TR) = 1.838 s, flip angle = 75 °, field of view = 220 × 220 mm2, matrix = 72 × 72, 32 slices, slice thickness = 3 mm, and 1 mm interslice gap; 700-s acquisition time, resulting in 350 functional volumes). The anatomical scan was acquired before the functional scan using a T1-weighted MPRAGE sequence with 240 × 240 × 170 voxels (1 × 1 × 1 mm voxel size) covering the whole brain. A total of 16 volunteers completed the full protocol and were included in our analyses; one participant was excluded due to high motion, leaving N=15 for analysis (31).

#### Functional MRI preprocessing and denoising

We applied a standard preprocessing pipeline in accordance with our previous publications with anaesthesia data (64, 141). Preprocessing was performed using the *CONN* toolbox, version 17f (CONN; http://www.nitrc.org/projects/conn) (143), implemented in MATLAB 2016a. The pipeline involved the following steps: removal of the first 10s, to achieve steady-state magnetization; motion correction; slice-timing correction; identification of outlier volumes for subsequent scrubbing by means of the quality assurance/artifact rejection software *art* (http://www.nitrc.org/projects/artifact_detect); normalisation to Montreal Neurological Institute (MNI-152) standard space (2 mm isotropic resampling resolution), using the segmented grey matter image from each participant’s T1-weighted anatomical image, together with an a priori grey matter template.

Denoising was also performed using the CONN toolbox, using the same approach as in our previous publications with pharmaco-MRI datasets (64, 141). Pharmacological agents can induce alterations in physiological parameters (heart rate, breathing rate, motion) or neurovascular coupling. The anatomical CompCor (aCompCor) method removes physiological fluctuations by extracting principal components from regions unlikely to be modulated by neural activity; these components are then included as nuisance regressors (144). Following this approach, five principal components were extracted from white matter and cerebrospinal fluid signals (using individual tissue masks obtained from the T1-weighted structural MRI images) (143); and regressed out from the functional data together with six individual-specific realignment parameters (three translations and three rotations) as well as their first-order temporal derivatives; followed by scrubbing of outliers identified by ART, using Ordinary Least Squares regression (143). Finally, the denoised BOLD signal time-series were linearly detrended and band-pass filtered to eliminate both low-frequency drift effects and high-frequency noise, thus retaining frequencies between 0.008 and 0.09 Hz. The step of global signal regression (GSR) has received substantial attention in the literature as a denoising method (145–147). However, recent work has demonstrated that the global signal contains behaviourally relevant information (148) and, crucially, information about states of consciousness, across pharmacological and pathological perturbations (24). Therefore, in line with ours and others’ previous studies, here we avoided using GSR to denoise our human fMRI data in favour of the aCompCor denoising procedure, which is among those recommended.

Finally, denoised BOLD signals were parcellated into 100 cortical regions-of-interest (ROIs) from the Schaefer atlas (149).

### Human Connectome Project data

We used resting-state functional MRI data from the 100 unrelated subjects (54 females and 46 males, mean age = 29.1 ± 3.7 years) of the HCP 900 subjects data release (150). All HCP scanning protocols were approved by the local Institutional Review Board at Washington University in St. Louis. The diffusion-weighted imaging (DWI) acquisition protocol is covered in detail elsewhere (151). Data were acquired using the following parameters. Structural MRI: 3D MPRAGE T1-weighted, TR = 2,400 ms, TE = 2.14 ms, TI = 1,000 ms, flip angle = 8°, FOV = 224 × 224, voxel size = 0.7 mm isotropic. Two sessions of 15-min resting-state fMRI: gradient-echo EPI, TR = 720 ms, TE = 33.1 ms, flip angle = 52°, FOV = 208×180, voxel size = 2 mm isotropic. Here, we used functional data from only the first scanning session, in LR direction.

We also used diffusion MRI (dMRI) data from the same 100 unrelated participants. The diffusion MRI scan was conducted on a Siemens 3T Skyra scanner using a 2D spin-echo single-shot multiband EPI sequence with a multi-band factor of 3 and monopolar gradient pulse. The spatial resolution was 1.25 mm isotropic. TR=5500 ms, TE=89.50ms. The b-values were 1000, 2000, and 3000 s/mm^2^. The total number of diffusion sampling directions was 90, 90, and 90 for each of the shells in addition to 6 b0 images. We used the version of the data made available in DSI Studio-compatible format at http://brain.labsolver.org/diffusion-mri-templates/hcp-842-hcp-1021 (152).

#### Functional MRI preprocessing and denoising

HCP-minimally preprocessed data (151) were used for all acquisitions. The minimal preprocessing pipeline includes bias field correction, functional realignment, motion correction, and spatial normalisation to Montreal Neurological Institute (MNI-152) standard space with 2mm isotropic resampling resolution (151).

We used the same denoising procedure as for the human anaesthesia dataset, based on the aCompCor method implemented in CONN (143).

### Macaque fMRI anaesthesia datasets

The macaque fMRI datasets used here have been previously reported; for clarity and consistency of reporting, where possible we use the same wording as in these previous reports, and we refer the reader to them for details (15, 22, 32).

#### Animals and ethics

For the macaque Multi-Anaesthesia dataset, N=5 rhesus macaques were included for analyses (*Macaca mulatta*, one male, monkey J, and four females, monkey A, K, Ki, and R, 5–8 kg, 8–12 yr of age), in a total of six different arousal conditions: awake state, deep ketamine, light propofol, deep propofol, light sevoflurane, and deep sevoflurane anaesthesia. Here, we used the awake, deep propofol, deep sevoflurane, and ketamine data. Three monkeys were used for each condition: awake state (monkeys A, K, and J), ketamine (monkeys K, R and Ki), propofol (monkeys K, R, and J), sevoflurane (monkeys Ki, R, and J). Each monkey had fMRI restingstate acquisitions on different days and several monkeys were scanned in more than one experimental condition. Sex was not considered in this study. Because of the small sample sizes, the sex balance per group could not be secured. All procedures are in agreement with the European Convention for the Protection of Vertebrate Animals used for Experimental and Other Scientific Purposes (Directive 2010/63/EU) and the National Institutes of Health’s Guide for the Care and Use of Laboratory Animals. Animal studies were approved by the institutional Ethical Committee (Commissariat a l’Energie atomique et aux Énergies alternatives; France; protocols CETEA 10–003 and 12–086). Additional details of the acquisitions can be found in the original publications (11, 15).

For the macaque DBS dataset, N=5 male rhesus macaques (*Macaca mulatta*, 9 to 17 years and 7.5 to 9.1 kg) were included, three for the awake (non-DBS) experiments (monkeys B, J, and Y) and two for the DBS experiments (monkeys N and T). Only males were included in the DBS dataset in order to avoid the menstrual cycle and hormone variations. All procedures are in agreement with 2010/63/UE, 86–406, 12–086 and 16–040. For additional details, we refer the reader to the original publication (32).

#### Anaesthesia protocol

For the Multi-Anaesthesia dataset, monkeys received anaesthesia either with ketamine, propofol, or sevoflurane (11, 15), with two different levels of anaesthesia for propofol and sevoflurane anaesthesia (light and deep). The anaesthesia levels were defined according to the monkey sedation scale (Table S5), based on spontaneous movements and the response to external stimuli (presentation, shaking or prodding, toe pinch), and corneal reflex (15) and EEG. EEG data were simultaneously acquired during MRI scanning (though they are not analysed in the present study). For each scanning session, the clinical score was determined at the beginning and end of the scanning session, together with continuous visual monitoring of electroencephalography monitoring. Monkeys were intubated and ventilated (11, 15). Heart rate, noninvasive blood pressure, oxygen saturation, respiratory rate, end-tidal carbon dioxide, and cutaneous temperature were monitored (Maglife, Schiller, France) and recorded online (Schiller).

During deep ketamine, deep propofol, and deep sevoflurane anaesthesia, monkeys stopped responding to all stimuli, reaching a state of general anaesthesia. For ketamine anesthesia, ketamine was injected intramuscular (20 mg/kg; Virbac, France) for induction of anesthesia, followed by a continuous intravenous infusion of ketamine (15 to 16 mg · kg^−1^ · h^−1^) to maintain anesthesia. Atropine (0.02 mg/kg intramuscularly; Aguettant, France) was injected 10 min before induction, to reduce salivary and bronchial secretions. For propofol anesthesia, monkeys were trained to be injected an intravenous propofol bolus (5 to 7.5 mg/kg; Fresenius Kabi, France), followed by a target-controlled infusion (Alaris PK Syringe pump, CareFusion, USA) of propofol (light propofol sedation, 3.7 to 4.0 *μ*g/ml; deep propofol anesthesia, 5.6 to 7.2 *μ*g/ml) based on the Paedfusor pharmacokinetic model. During sevoflurane anesthesia, monkeys received first an intramuscular injection of ketamine (20 mg/kg; Virbac) for induction, followed by sevoflurane anesthesia (light sevoflurane, sevoflurane inspiratory/expiratory, 2.2/2.1 volume percent; deep sevoflurane, sevoflurane inspiratory/expiratory, 4.4/4.0 volume percent; Abbott, France). Only 80 minutes after the induction, the scanning sessions started for the sevoflurane acquisitions to get a washout of the initial ketamine injection. To avoid artefacts related to potential movements throughout magnetic resonance imaging acquisition, a muscle-blocking agent was coadministered (cisatracurium, 0.15 mg/kg bolus intravenously, followed by continuous intravenous infusion at a rate of 0.18 mg · *kg*^−1^ · *h*^−1^; GlaxoSmithKline, France) during the ketamine and light propofol sessions. Here we include the ketamine, deep propofol, and deep sevoflurane data.

For the DBS dataset (32), anaesthesia was induced with an intramuscular injection of ketamine (10 mg/kg; Virbac, France) and dexmedetomidine (20 *μ*g/kg; Ovion Pharma, USA) and then the same method as reported above for deep propofol sedation was used (Monkey T: TCI, 4.6 to 4.8 *μ*g/ml; monkey N: TCI, 4.0 to 4.2 *μ*g/ml). Awake scanning data were obtained from the remaining three animals, who did not provide anaesthesia data.

#### Deep Brain Stimulation protocol

Two monkeys (N and T) were implanted with a clinical DBS electrode (Medtronic, Minneapolis, MN, USA, lead model 3389). The DBS lead had four active contacts for electrical stimulation (1.5-mm contact length, 0.5-mm spacing, and 1.27-mm diameter). We performed stereotactic surgery, targeting the right centromedian thalamus using a neuronavigation system (BrainSight, Rogue, Canada), guided by the rhesus macaque atlases (153, 154) and a preoperative and intraoperative anatomical MRI [MPRAGE (magnetization prepared - rapid gradient echo), T1-weighted, repetition time (TR) = 2200 ms, inversion time (TI) = 900 ms, 0.80-mm isotropic voxel size, and sagittal orientation]. The electrode was stabilized with the Stimloc lead anchoring device (Medtronic, Minneapolis, MN, USA). The extracranial part of the DBS lead was hosted using a homemade three-dimensional (3D) printed MRI-compatible chamber. We waited at least 20 days after implantation before starting the DBS-fMRI experiments. Two methods were used to ensure for the anatomical localization of the DBS lead and the DBS contacts. First, a reconstruction method based on in vivo brain imaging. Second, a histology study in one of the implanted monkeys (for more details, see (32)).

For stimulation, the DBS electrode was plugged to an external stimulator (DS8000, World Precision Instrument, USA), and all the parameters were tuned to a fixed value of frequency (f = 130.208 Hz, T = 7.68 ms), waveform (monopolar signal), and length of width pulse (monkey N, w = 320 *μ*s; monkey T, w = 140 *μ*s). The absolute voltage amplitude was set to 3V (‘low’ DBS) or 5V (‘high’ DBS). The resting-state fMRI experiments were acquired either in the awake state or under propofol anaesthesia without (‘off’ condition) or with a low or high DBS during the entire run on either the CT or VT thalamic nuclei. The DBS started a few seconds before the beginning of the fMRI sequence and stopped just after the end of the MRI sequence (32). Here, we included the awake and no-DBS conditions, as well as 5V CT and 5V VT stimulation conditions.

As reported by Tasserie et al., (32), ‘DBS significantly affected the general physiology parameters of the anaesthetized monkeys such as the mean heart rate (*P* = 3.38× 10^−26^) and mean blood pressure (*P* = 5.78× 10^−17^). For example, for monkey T, high CT-DBS significantly increased mean heart rate (*P* = 1.23× 10^−23^, compared to anaesthesia; *P* = 6.76× 10^−19^, compared to low CT-DBS condition) and mean blood pressure (*P* = 8.66× 10^−14^, compared to anaesthesia condition; *P* = 6.67× 10^−11^, compared to low CT-DBS condition)’.

#### Behavioural assessment of arousal

We used a preclinical behavioural scale adapted from **(author?)** (155) to assess the arousal levels of the monkeys. This scale, based on the Human Observers Assessment of Alertness and Sedation Scale (156) and previously utilised in non-human primate (NHP) research (157), was used consistently across all experimental conditions, in both datasets. The arousal testing occurred outside the MRI environment and was conducted at the beginning and end of each scanning session, for each condition, once the animals were no longer under paralysis. The assessment encompassed six criteria as described in S5. The behavioural score ranged from 0 to 11, where 11 represented the maximum score achievable and 0 the lowest.

In all cases, and for both datasets, we observed no differences in arousal scores between different animals in the same condition. For both datasets, the behavioural score during wakefulness was the maximum of 11/11 for all the animals (Monkey A, Monkey K, and Monkey J from the Multi-Anaesthesia dataset, and Monkeys B, J and Y from the DBS dataset): exploration of the surrounding world = 2; spontaneous movements = 2; shaking/prodding = 2; toe pinch = 2; eyes opening = 2; corneal reflex = 1.

For results pertaining to the different anaesthesia conditions of the Multi-Anaesthesia dataset, see Table S25 of (22). As a summary, deep anaesthesia with ketamine, propofol, or sevoflurane induced an arousal score of 0, consistently in all animals. In contrast, light anaesthesia induced an arousal score of 3 for sevoflurane, and 4 for propofol.

In the anesthesia without DBS (‘off’) condition, monkeys N and T displayed the minimum behavioral score of 0 over 11, same as the deep anaesthesia from the Multi-Anaesthesia dataset: exploration of the surrounding world = 0; spontaneous movements = 0; shaking/prodding = 0; toe pinch = 0; eyes opening = 0; corneal reflex = 0. When the CT electrical stimulation amplitude was increased to 5V (high-amplitude CT DBS), animals reached a total score of 9 over 11 (exploration of the surrounding world = 1; spontaneous movements = 1; shaking/prodding = 2; toe pinch = 2; eyes opening = 2; corneal reflex = 1). For VT DBS, both low (3V) and high (5V) amplitude stimulation led to a clinical score of 0, identical to what is observed in the absence of any stimulation.

#### MRI data acquisition

For both Multi-Anaesthesia and DBS datasets, for the awake condition, monkeys were implanted with a magnetic resonance compatible head post and trained to sit in the sphinx position in a primate chair (158). For the awake scanning sessions, monkeys sat inside the dark magnetic resonance imaging scanner without any task and the eye position was monitored at 120 Hz (Iscan Inc., USA). The eye-tracking was performed to make sure that the monkeys were awake during the whole scanning session and not sleeping. The eye movements were not regressed out from rfMRI data. For the anesthesia sessions, animals were positioned in a sphinx position, mechanically ventilated, and their physiologic parameters were monitored. No eye-tracking was performed in anesthetic conditions.

For the Multi-Anesthesia dataset, before each scanning session, a contrast agent, monocrystalline iron oxide (MION) nanoparticle (Feraheme, AMAG Pharmaceuticals, USA; 10 mg/kg, intravenous), was injected into the monkey’s saphenous vein (159). Monkeys were scanned at rest on a 3-Tesla horizontal scanner (Siemens Tim Trio, Germany) with a single transmit-receive surface coil customised to monkeys. Each functional scan consisted of gradient-echo planar whole-brain images (repetition time = 2,400 ms; echo time = 20 ms; 1.5-mm3 voxel size; 500 brain volumes per run).

For the DBS dataset, monkeys were scanned at rest on a 3-Tesla horizontal scanner (Siemens, Prisma Fit, Erlanger Germany) with a customised eight-channel phased-array surface coil (KU Leuven, Belgium). The parameters of the functional MRI sequences were: echo planar imaging (EPI), TR = 1250 ms, echo time (TE) = 14.20 ms, 1.25-mm isotropic voxel size and 500 brain volumes per run. Event-related data pertaining to auditory stimulation were also acquired and are reported in (32), but here we only used the resting-state fMRI data, and will not discuss the event-related data further. Scalp EEG data were also acquired using an MR-compatible system and custom-built caps (EasyCap, 13 channels), an MR amplifier (BrainAmp, Brain Products, Germany), and the Vision Recorder software (Brain Products). These results are reported in (32), but here we did not consider the EEG data and will not discuss them further.

#### Macaque functional MRI preprocessing, denoising, and time-series extraction

For the Multi-Anaesthesia dataset, a total of 157 functional magnetic imaging runs were acquired (15): Awake, 31 runs (monkey A, 4 runs; monkey J, 18 runs; monkey K, 9 runs), Ketamine, 25 runs (monkey K, 8 runs; monkey Ki, 7 runs; monkey R, 10 runs), Light Propofol, 25 runs (monkey J, 2 runs; monkey K, 11 runs; monkey R, 12 runs), Deep Propofol, 31 runs (monkey J, 9 runs; monkey K, 10 runs; monkey R, 12 runs), Light Sevoflurane, 25 runs (monkey J, 5 runs; monkey Ki, 10 runs; monkey R, 10 runs), Deep Sevoflurane anaesthesia, 20 runs (monkey J, 2 runs; monkey Ki, 8 runs; monkey R, 10 runs). Additional details are available from the original publications (11, 15, 75).

Functional images were reoriented, realigned, and rigidly coregistered to the anatomical template of the monkey Montreal Neurologic Institute (Montreal, Canada) space with the use of Python programming language and FMRIB Software Library (FSL) software (http://www.fmrib.ox.ac.uk/fsl/; accessed February 4, 2018) (15). From the images, the global signal was regressed out to remove confounding effect due to physiologic changes (e.g., respiratory or cardiac changes).

For the DBS dataset, a total of 199 Resting State functional MRI runs were acquired: Awake 47 runs (monkey B: 18 runs; monkey J: 13 runs; monkey Y: 16 runs), anaesthesia (DBS-off) 38 runs (monkey N: 16 runs,; monkey T: 22 runs), low amplitude centro-median thalamic DBS 36 runs (monkey N: 18 runs; monkey T: 18 runs), low amplitude ventro-lateral thalamic DBS 20 runs (monkey T), high amplitude centro-median thalamic DBS 38 runs (monkey N: 17 runs; monkey T: 21 runs), and high amplitude ventro-lateral thalamic DBS 20 runs (monkey T: 20 runs) (32).

Images were preprocessed using Pypreclin (Python preclinical pipeline) (10). Functional images were corrected for slice timing and B0 inhomogeneities, reoriented, realigned, resampled (1.0 mm isotropic), masked, coregistered to the MNI macaque brain template (160), and smoothed (3.0-mm Gaussian kernel). Anatomical images were corrected for B1 inhomogeneities, normalised to the anatomical MNI macaque brain template, and masked.

For both datasets, data were parcellated according to the Regional Map parcellation (129). This parcellation comprises 82 cortical ROIs (41 per hemisphere). Voxel time series were filtered with low-pass (0.05-Hz cutoff) and high-pass (0.0025-Hz cutoff) filters and a zero-phase fast-Fourier notch filter (0.03 Hz) to remove an artifactual pure frequency present in all the data (11, 15, 32).

Furthermore, an extra quality control (QC) procedure was performed to ensure the quality of the data after time-series extraction (75). This quality control procedure is based on trial-by-trial visual inspection by an expert neuroimager (C.M.S.), and it is the same as was previously implemented in (22, 75). Its adoption ensures that we employ consistent criteria across our two macaque datasets, by adopting the more stringent of the two. We plotted the time series of each region, as well as the static functional connectivity matrix (FC), the dynamic connectivity (dFC) and a Fourier analysis to detect unconventional spikes of activity. For each dataset, visual inspection was first used to become familiar with the characteristics of the entire dataset: how the amplitude spectrum, time-series, FC and dynamic FC look. Subsequently, each trial was inspected again with particular focus on two main types of potential artefacts. The first one may correspond to issues with the acquisition and is given by stereotyped sinusoidal oscillatory patterns without variation. The second one may correspond to a head or other movement not corrected properly by our preprocessing procedure. This last artefact can be sometimes recognized by bursts or peaks of activity. Sinusoidal activity generates artificially high functional correlation and peak of frequencies in the Amplitude spectrum plot. Uncorrected movements generate peaks of activity with high functional correlation and sections of high functional correlations in the dynamical FC matrix. If we observed any of these anomalies we rejected the trial, opting to adopt a conservative policy. See Figures S19–S21 from (22) for examples of artifact-free and rejected trials.

As a result, for the Multi-Anaesthesia data set a total of 119 runs are analysed in subsequent sections (the same as used in (75)): awake state 24 runs, ketamine anaesthesia 22 runs, light propofol anaesthesia 21 runs, deep propofol anaesthesia 23 runs, light sevoflurane anaesthesia 18 runs, deep sevoflurane anaesthesia 11 runs. For the DBS data set, a total of 156 runs are analysed in subsequent sections: awake state 36 runs, Off condition (propofol anaesthesia without stimulation) 28 runs, low-amplitude CT stimulation 31 runs, low-amplitude VT stimulation 18 runs, high-amplitude CT stimulation 25 runs, high-amplitude VT stimulation 18 runs.

### Marmoset fMRI anaesthesia dataset

The marmoset data included here have been published before (34). For clarity and consistency of reporting, we use the same wording as in the original publication where possible, and we refer the reader to the original work for details (34).

#### Animals and ethics

This study was approved by the Animal Experiment Committees at the RIKEN Center for Brain Science (CBS) and was conducted per the guidelines for Conducting Animal Experiments of RIKEN CBS. Three male and one female healthy common marmosets (*C. jacchus*) between 3 and 6 years of age were included. All marmosets were examined 8 times to collect functional MRI data in all conditions. After awake data were firstly collected, and sedate/anesthetic data were done in a random order for sedate/anesthetic condition with an interval of 1 month between each examination in each individual. For further details we refer the reader to the original publication (161).

#### Anesthesia protocol

We used data from awake scans, and from scans under general anaesthesia induced with isoflurane, sevoflurane, or propofol. Essential details are reported below, and we refer to the original publication by **(author?)** (34) for additional detail.

##### Isoflurane.

Three percent isoflurane with 100% O2 as carrier gas was administered through a facial mask to the marmosets retained with leather gloves. Once sufficient sedation was achieved, an 8 Fr catheter (Atom multi-use tube, Atom Medical Corp., Tokyo, Japan) was inserted into the trachea as an intratracheal tube. The isoflurane concentration was reduced to 2.5%, and 50 *μ*g/kg of atropine and 3.0 mL of physiological saline were administered subcutaneously to prevent intratracheal secretion and dehydration, respectively. After intratracheal intubation, general anesthesia was maintained with 1.8% isoflurane. The marmosets were connected to artificial ventilation for small animals (SN-480-7, Shinano Seisakusho, Tokyo, Japan), and mechanical ventilation was performed under the following conditions: 50% inspiratory oxygen, 8 mL of tidal volume, and 30 breaths per min RR. PR, SpO2, RR, EtCO2, inspiratory and end-tidal isoflurane concentrations, and rectal temperature were measured with a vital sign monitor.

##### Sevoflurane.

All procedures were performed in the same way as isoflurane, except for dosages. The induction of general anesthesia, intratracheal intubation, and maintenance of general anesthesia were performed with 5.0 and 3.0% of sevoflurane (Pfizer Japan Inc., Tokyo, Japan), respectively.

##### Propofol.

Propofol (12 mg/kg) was administered as induction of general anesthesia over 3 min via an indwelling needle. Atropine (50 *μ*g/kg) was administered subcutaneously to prevent intratracheal secretion. Immediately after bolus administration, the predicted plasma concentration of propofol was titrated to 7–9 *μ*g/mL for intratracheal intubation with continuous infusion. The administration dose and protocol were calculated beforehand using a pharmacokinetic parameter reported by (161) and pharmacokinetic analysis software, NONMEM ver. VII (GloboMax ICON Development Solutions, Ellicott City, MD, USA). Once a sufficient plasma concentration was obtained, the propofol dose was controlled to maintain that concentration. Intratracheal intubation, respiratory management, and vital sign monitoring were performed in the same manner as for isoflurane.

#### MRI data acquisition

During imaging, the marmosets were placed on a custom-made imaging table (Takashima Seisakusho Co., Ltd, Tokyo, Japan) and immobilized by fixing the head post using a head post fixing tool attached at a custom-made imaging table in all conditions. The marmosets were fitted with earplugs. A hot water circulator was used during imaging to maintain body temperature 36–38 °C under all conditions (34).

In Awake condition, data were collected in the dark and monitored with an infrared camera to prevent the marmosets from falling asleep. If the marmosets were observed closing their eyes during a scan, they were awakened with a loud noise before the next scan was started. They were rewarded with a highly palatable food at the end of each imaging. In anesthetic condition, the marmosets were monitored in the same way to observe spontaneous movement or coughing for intratracheal tube. If spontaneous movement or coughing was observed, additional sedatives/anesthetics were administrated and infusion rate or concentration of inhalational anesthesia was increased.

An ultra-high field MRI system with a static magnetic field strength of 9.4 T (Bruker BioSpin, Ettlingen, Germany), a custom-made 8-channel receiver coil for the marmoset head (Takashima Seisakusho Co., Ltd, Tokyo, Japan), and a 154 mm inner diameter transmitter coil (Bruker BioSpin, Ettlingen, Germany) were used to collect structural and functional data. Structural data and T2-weighted images were imaged using rapid acquisition with relaxation enhancement (RARE) sequence with the following conditions and parameters: time repetition (TR)=4331 ms, time echo (TE) = 15.0 ms, FOV = 42.0 × 28.0 × 36.0 mm, matrix size = 120 × 80 voxels, resolution = 0.35 × 0.35 mm, slice thickness = 0.7 mm, number of slices = 52, scan time = 1 min and 26 s, RARE factor = 4. Functional images were captured using a gradient recalled echo-planar imaging (EPI) sequence with the following conditions and parameters: TR = 2,000 ms, TE = 16.0 mm, FOV = 42.0 × 28.0 × 36.0, mm matrix size = 60 × 40 voxels, resolution = 0.7 × 0.7 mm, slice thickness = 0.7 mm, number of slices = 52, repetition = 155, scan time = 310s. Functional imaging was performed 12 times per animal, per condition (34).

#### Marmoset functional MRI preprocessing and denoising

After the acquired data were converted to Neuro Informatics Technology Initiative format (NIfTI), the voxel size was changed from 0.7 mm isotropic to 3.5 mm isotropic using SPM (Wellcome Trust Center for Neuroimaging, London, UK). Estimation and correction of geometric distortions induced by magnetic susceptibility were performed with the top-up tool of the FMRIB Software Library (FSL) software (FMRIB, Oxford, UK) because all cross-sections were imaged with a single excitation in EPI. Slice timing correction was performed to correct for signal acquisition timing discrepancies in each section. Realignment was applied to compensate for head movements caused by body movements. The deviations in 6 directions were obtained: x (left/right), y (front/back), z (up/down), pitch (rotational direction of nodding and looking up), roll (rotational direction of moving the ear closer to the shoulder), and yaw (rotational direction of looking left/right). For each measurement time point (TR), the deviation from the reference time point, and the first functional brain image, was determined; and the image was moved and rotated by the rigid body model based on this deviation. The method of finding the parameters of the linear transformation was used to minimize the difference between the first functional brain image and the affine transformation of the series of functional brain images to be corrected, by calculating convergence using the method of least squares. After correcting the spatial scale error between the structural and functional images with co-registration, segmentation was performed to provide information on the tissue to which each voxel belongs in terms of brain tissue classification. The voxels were spatially standardized by normalization, which aligns the voxels to the standard brain image to correct for structural differences between individuals. Smoothing was applied to suppress excessive voxel value fluctuations within individuals and apply normal probability field theory. Functional data were smoothed using spatial convolution with a Gaussian kernel of 2 voxels (7 mm). Then, physiological noise was denoised using ordinary least squares regression with cerebrospinal fluid pulsation, heart rate, and respiratory artifacts as regressors. Temporal band pass filtering was performed by frequency filtering (0.01–0.1 Hz) using the fMRI denoising pipeline of CONN (143). Finally, preprocessed functional data were parcellated into 70 cortical regions from the marmoset MBM-vM atlas (162).

### Mouse fMRI anaesthesia dataset

The mouse fMRI data used here have been published before (14). For clarity and consistency of reporting, where possible we use the same wording as in the original publication (14).

#### Animals and ethics

In vivo experiments were conducted in accordance with the Italian law (DL 26/214, EU 63/2010, Ministero della Sanita, Roma) and with the National Institute of Health recommendations for the care and use of laboratory animals (14). The animal research protocols for this study were reviewed and approved by the Italian Ministry of Health and the animal care committee of Istituto Italiano di Tecnologia (IIT). All surgeries were performed under anesthesia.

Adult (< 6 months old) male C57BL/6J mice were used throughout the study. Mice were group housed in a 12:12 hours light-dark cycle in individually ventilated cages with access to food and water ad libitum and with temperature maintained at 21 ± 1 degrees centigrade and humidity at 60 ± 10%. All the imaged mice were bred in the same vivarium and scanned with the same MRI scanner and imaging protocol employed for the awake scans (see below).

A first group of mice (n = 10, awake dataset) underwent head-post surgery, scanner habituation and fMRI image acquisitions as described below. See (14) for the full surgical, habituation, and scanner protocol. The scans so obtained constitute the awake rsfMRI mouse dataset we used throughout our study. Two additional groups of age matched male C57BL/6J mice were used as reference rsfMRI scans under anesthesia.

#### Anaesthesia protocol

The first group of animals (n = 19, halothane dataset) was previously scanned under shallow halothane anaesthesia, 0.75% (83). The employed anaesthesia regimen is well characterized (14, 163); it is representative of the network architecture observed with different anaesthesia regimens in rodents (84) and it exhibits rich spatiotemporal dynamics by preserving spectral properties of fMRI signal fluctuations (83).

A second, separate group (n = 14) of mice were imaged under medetomidine-isoflurane anesthesia (0.05 mg/kg bolus and 0.1 mg/kg/h IV infusion, plus 0.5% isoflurane) (14). While this anesthetic combination is known to shift the spectral components of fMRI signal fluctuations towards higher frequencies (hence departing from the characteristic 1/f power law distribution that characterizes awake and halothane rsfMRI datasets (83, 84)), it nonetheless represents the mostwidely anesthetic mixture used in the rodent imaging community (84).

#### MRI data acquisition

For awake scanning, the mouse was secured using an implanted headpost the custom-made MRI-compatible animal cradle and the body of the mouse was gently restrained (for details of the headpost implantation and habituation protocol, see the original publication (14)). For scanning under anesthesia, mice were first deeply anesthetized with isoflurane (4% induction), intubated and artificially ventilated (90 BPM). In one group, anesthesia was then switched to halothane (0.75%). In a second group, a bolus of medetomidine (0.05 mg/kg) was given via tail vein cannulation before waiting 5 minutes and starting an infusion of medetomidine (0.1 mg/kg/h) with isoflurane reduced to 0.5%. In both cases, the rsfMRI acquisition started 30 minutes after the switch to light anesthesia (14).

All scans were acquired at the IIT laboratory in Rovereto (Italy) on a 7.0 Tesla MRI scanner (Bruker Biospin, Ettlingen) with a BGA-9 gradient set, a 72 mm birdcage transmit coil, and a four-channel (awake, halothane) or three-channel (medetomidine-isoflurane) solenoid receive coil. Awake and medetomidine-isoflurane rsfMRI scans were acquired using a single-shot echo planar imaging (EPI) sequence with the following parameters: TR/TE=1000/15 ms, flip angle=60 degrees, matrix=100 × 100, FOV=2.3 × 2.3 cm, 18 coronal slices (voxel-size 230 × 230 × 600 mm), slice thickness=600 mm and 1920 time points, for a total time of 32 minutes. Mice under halothane anesthesia (n = 19) were scanned with a TR/TE=1200/15ms, flip angle=60 degrees, matrix=100 × 100, 24 coronal slices (voxel-size 200 × 200 × 500 mm), for a total of 1600 time points, total acquisition time of 32 minutes as described in (14).

#### Mouse functional MRI preprocessing and denoising

Preprocessing of fMRI images was carried out as described in previous work (14). Briefly, the first 2 minutes of the time series were removed to account for thermal gradient equilibration. Functional MRI time-series were then time despiked (3dDespike, AFNI), motion corrected (MCFLIRT, FSL), skull stripped (FAST, FSL) and spatially registered (ANTs registration suite) to an inhouse mouse brain template with a spatial resolution of 0.23 × 0.23 × 0.6 mm^3^. Denoising involved the regression of 25 nuisance parameters. These were: average cerebral spinal fluid signal plus 24 motion parameters determined from the 3 translation and rotation parameters estimated during motion correction, their temporal derivatives and corresponding squared regressors. No global signal regression was employed. In-scanner head motion was quantified via calculations of frame-wise displacement (FD). Average FD levels in awake conditions were comparable to those obtained in anesthetized animals (halothane) under artificial ventilation (p = 0.13, Student t-test) (14). To rule out a contribution of residual head-motion, we further introduced frame-wise fMRI scrubbing (FD > 0.075 mm). The resulting time series were band-pass filtered (0.01–0.1 Hz band) and then spatially smoothed with a Gaussian kernel of 0.5 mm full width at half maximum. Finally, the time-series were trimmed to ensure that the same number of timepoints were included for all animals, resulting in 1414 volumes per animal. Finally data were parcellated into 72 cortical symmetric regions from the Allen Mouse Brain Atlas (CCFv3).

### Nematode calcium imaging dataset

The *C. elegans* data included in this study have been published before. For clarity and consistency of reporting, where possible we use the same wording as in the original publications (33, 35).

#### C. elegans strains

All experiments were on young adult hermaphrodites of the transgenic strain QW1217 (zfIs124[Prgef1::GCaMP6s]; otIs355[Prab-3::NLS::tagRFP]). GCaMP6s, a fluorescent calcium reporter, and nuclear-localized red fluorescent protein are both expressed pan-neuronally in this strain (gift of M. Alkema, University of Massachusetts, Worcester, Massachusetts). *C. elegans* were cultivated at 20 °C on nematode growth medium seeded with Escherichia coli OP50 (35).

#### Anaesthesia protocol

*C. elegans* become anesthetized on exposure to isoflurane with a minimum alveolar concentration (MAC) value of approximately 3% at room temperature. At 17 °C, isoflurane is 2.3 times more soluble in tissues such as muscle and fat than at 37°C,21 and consequently absorption of a larger quantity of isoflurane is required to produce a similar chemical potential gradient at cooler temperatures. A concentration of 3% isoflurane at 17 °C is thus pharmacodynamically similar, with respect to its physical chemistry, to a concentration of 1.3% at 37 °C. Alterations in *C. elegans* neuronal activity in response to isoflurane exposure were assessed using two experimental regimes: stepwise anesthetization and emergence (35). Here we include data from the stepwise anesthetisation regime.

Three 5-min-long neuronal activity recordings were taken from each animal (n = 10) after progressive equilibration to 0%, 4%, and 8% atmospheric isoflurane (35). These concentrations correspond to 1.3 and 2.6 MAC, respectively. For our main analysis we include data from the 0% and 4% isoflurane conditions. Imaging was performed at time t = 30 min, 150 min, and 270 min from the start of the experiment at isoflurane levels of 0%, 4%, and 8%. Isoflurane levels were stepped by exchanging the immersion medium for fresh S-basal buffer with the addition of 13 or 26 *μ*l of pipetted isoflurane for 4% and 8% isoflurane, respectively. The atmosphere within the covered Petri dish was then equilibrated, during the time between imaging sequences, to a concentration of 4% or 8%, respectively, using continuous monitoring with an infrared spectrometer (Ohmeda 5250 RGM; GE Healthcare, USA) and instillation of isoflurane via syringe pump as necessary to maintain the targeted concentration (35).

#### Calcium imaging of neuronal activity

GCaMP6s and red fluorescent protein fluorescence in the *C. elegans* head ganglia were captured in volumetric stacks using a dual inverted selective plane illumination microscope (Applied Scientific Instrumentation, USA) and water-immersed 0.8 NA 40x objective (Nikon, USA). GCaMP6s and red fluorescent protein were respectively excited using 5-mW 488-nm and 561-nm lasers (Vortan Laser Technology, USA). Volumes for each fluorescent channel (GCaMP6s and red fluorescent protein) were obtained at rate of 2 Hz. The animals were immobilized for imaging by encapsulation in a pad of permeable hydrogel consisting of 13.3% polyethylene glycol diacrylate (Advanced BioMatrix, USA) with 0.1% Irgacure (Sigma-Aldrich, USA) (35). Hydrogel pads containing animals to be imaged were cured with ultraviolet light onto silanated glass coverslips, which were affixed to the bottom of 50-mm Petri dishes with vacuum grease. Petri dishes were then filled with 50 mL of S-basal buffer (100 mM NaCl, 50 mM KPO4 buffer, and 5 *μ*g/ml cholesterol) as the immersion medium. Tetramisole was added to this buffer at 5 mM to further immobilize the animals.

For each animal imaged, 120 neurons (ordered from anterior to posterior in each animal) were tracked in the head region using the nuclear-localized red fluorescent protein fluorophore, and their activity was extracted using the fluctuations in cytoplasmic GCaMP6s neurofluorescence. This tracking and extraction procedure was performed as a massively parallel computation executed at the Massachusetts Green High Performance Computing Center using computational techniques as previously detailed by (33).

#### Preprocessing of neuronal activity traces

In recordings from the stepwise equilibration regime, neuronal activity intensity for all three recordings in each animal (0%, 4%, and 8%) was normalized against the average intensity across all neurons and time points in the 0% isoflurane recording. The first 100 time-points were excluded to remove large transients, leaving 500 for analysis. Because the animals are immobilized and encapsulated in a hydrogel for imaging, it is not possible to normalize to an alternative behavioral endpoint (35).

### Massive temporal feature extraction using hctsa

To extract the dynamical phenotype of each brain region or neuron, we performed massive time-series feature extraction using the *highly comparative timeseries analysis* toolbox, hctsa (28, 29). For each region/neuron, in each individual, under each condition, the hctsa toolbox extracted >7 200 univariate dynamical features, derived from diverse fields including neuroscience, physics, ecology and economics (28, 29). Features range from basic statistics of the distribution of time-points, linear correlations among time-points, and stationarity, to measures of entropy, time-delay embeddings, and signal complexity, among others. Each individual feature is the implementation of a computation (termed ‘master operation’) on the input time-series, using specific parameters. For example, sample entropy (SampEn) computes the probability that similar sequences of observations in a time-series will remain similar as their size increases. Its computation therefore requires a threshold *r* for deciding when two sequences will be considered similar; and an embedding dimension *m* that determines the size of the sequences. Multiple individual features are obtained from this master operation as different combinations of *r* and *m*.

We performed an initial pre-filtering, and did not compute any features that had returned *NaN* across all regions, or that had displayed no variance, in an independent dataset of human functional MRI (Human Connectome Project; (150)). The values of different features can vary across several orders of magnitude. Unless otherwise specified, here we do not normalise features across regions, since this could obscure differences across conditions. Instead, we use effect sizes computed on the original features (see below). Despite our initial pre-filtering, not all of the remaining time-series features could be extracted successfully from all our datasets. We therefore performed an additional post-filtering. Features that failed to be extracted were excluded. To ensure consistency, features were only included in the final analysis if they could be included for each dataset. Features that failed to produce a finite effect size for a region, had their effect size for that region set to zero. Following pre-filtering and post-filtering, a total of 6 958 dynamical features were retained across datasets.

hctsa provides features pertaining to a broad variety of categories, such as distribution, correlation, information theory, stationarity, and scaling. Our final set includes 18 broadly-defined categories: autocorrelation, correlation, distribution, entropy, forecast, information theory, nonlinear, outliers, preprocessing, scaling, spectrum, stationarity, statistics, surrogate, symbolic, trend, wavelet, and a final category of ‘other’ that could not be otherwise classified.

#### Dynamical profile similarity

To quantify the similarity between the temporal profiles of each pair of brain regions (neurons in the nematode), each dynamical feature is z-scored across regions. The vectors of z-scored regional features are then correlated for each pair of brain regions, producing a matrix of ‘dynamical profile similarity’ (also termed ‘temporal profile similarity’ (30)) that represents the strength of the similarity of the local dynamical fingerprints of brain areas. This procedure is performed separately within each individual and condition of each dataset.

The coupling between DPS and functional connectivity is in turn obtained by correlating the vectorised DPS and FC matrices. FC is computed as the zero-lag correlation between pairs of regional time-series.

#### catch22 subset of representative features

Among all hctsa dynamical features, **(author?)** (47) identified a reduced set of 22 features that captures a diverse range of interpretable time-series properties from the broader literature on dynamical systems (S2). The 22 features in this subset exhibit strong classification performance across a broad collection of time-series problems, with minimal redundancy and drastically reduced computation time **(author?)** (47). We use this reduced set for our multivariate association analysis with regional gene expression, and as the fitting target for our computational model. Note that one of these 22 features, *CO_HistogramAMI_even_2_5*, was excluded by our pre-filtering and therefore is not included among the features that we extract. A second feature, ‘acf timescale’ (first1e_acf_tau), occasionally failed to produce finite effect sizes for every region of every dataset due to no variance, and therefore had to be excluded from the PLS analysis. However, we still included this feature in the modelling analysis based on mean values, whereby non-finite values were ignored when computing the empirical results.

### Cortical gene expression

#### Brain-related genes

We obtained a list of 124 key genes pertaining to brain function from (45). This list includes receptor subunit genes, as well as cell-type markers for parvalbumin, somatostatin, vasoactive intestinal peptide, calbindin, and the four ‘most abundant mRNAs in myelin’ (45): *MBP*, *FTH1*, *PLEKHB1*, and *MOBP*. We augmented this list with receptor-related genes from (164, 165). We further added genes coding for *SLC6* transporters, histidine decarboxylase (*HDC*), and syntaxin (*STX1A*), hyperpolarization-activated cyclic nucleotide-gated channels (*HCN*), potassium channels (*KCN*), and sodium voltage-gated channels (*SCN*), which have been previously implicated in anaesthetic action (9, 20, 166). Of these, 81 genes were available and passed our quality control in all three species (human, macaque, and mouse), and were included in our analysis (Table S3 and Table S4).

#### Human brain gene expression from microarray

Regional human gene expression profiles were obtained using microarray data from the Allen Human Brain Atlas (AHBA) (42), with preprocessing as recently described (167). The Allen Human Brain Atlas (AHBA) is a publicly available transcriptional atlas containing gene expression data measured with DNA microarrays and sampled from hundreds of histologically validated neuroanatomical structures across normal postmortem human brains from six donors (five male and one female; age 24–55 years). We extracted and mapped gene expression data to the 100 cortical ROIs of the Schaefer parcellation using the abagen toolbox https://abagen.readthedocs.io/ (168). Data were pooled between homologous cortical regions to ensure adequate coverage of both left (data from six donors) and right hemisphere (data from two donors). Distances between samples were evaluated on the cortical surface with a 2mm distance threshold. Only probes where expression measures were above a background threshold in more than 50% of samples were selected. A representative probe for a gene was selected based on highest intensity. Gene expression data were normalised across the cortex using scaled, outlier-robust sigmoid normalisation. 15,633 genes survived these preprocessing and quality assurance steps. We also replicate our main results using human gene expression data from an alternative modality, RNA-seq, which was available from 2/6 AHBA donors (42).

#### Macaque cortical gene expression from stereo-seq

We used cortex-wide macaque gene expression data recently made available by (43), who combined single-nucleus RNA sequencing (“snRNA-seq”) with high-resolution, large-field-of view spatial transcriptomics from spatiotemporal enhanced resolution omicssequencing (“stereo-seq”) (43). Specifically, the authors made available (https://macaque.digital-brain.cn/spatial-omics) post-mortem gene expression data covering 143 regions of the left cortical hemisphere of one 6yo male cynomolgus macaque (*Macaca fascicularis*). We refer the reader to (43) for details. The animal protocol was approved by the Biomedical Research Ethics Committee of CAS Center for Excellence in Brain Science and Intelligence Technology, Chinese Academy of Sciences (ION-2019011). Animal care complied with the guideline of this committee (43).

Briefly, Chen and colleagues obtained 119 coronal sections at 500-*μ*m spacing, covering the entire cortex of the left hemisphere, which were used for stereo-seq transcriptomics (43). Adjacent 50-*μ*m thick sections were also acquired for regional microdissection and snRNA-seq analysis, as well as 10-*μ*m sections adjacent to each stereo-seq section, which were used for the anatomical parcellation of brain regions via immunostaining (43). Stereo-seq is a DNA nanoball (DNB) barcoded solidphase RNA capture method (43). It involves reverse transcription of RNAs released from frozen tissue sections fixated onto the stereo-seq chip, and subsequent PCR amplification. The resulting “amplified-barcoded complementary DNA (cDNA) is used as template for library preparation, and sequenced” to obtain high-resolution spatially resolved transcriptomics (43).

Gene expression data were made available for 143 cortical regions of the left hemisphere, including prefrontal, frontal, cingulate, somatosensory, insular, auditory, temporal, parietal, occipital and piriform areas. As reported in (43), for each coronal section, the cortical region and layer parcellation were manually delineated on Stereo-seq data background, based on cytoarchitectual pattern (e.g. cell density, cell size) revealed by total mRNA expression, nucleic acid staining, and NeuN staing of adjacent sections. To make the gene expression data compatible with our macaque functional MRI datasets, the aggregated gene expression across layers was manually mapped onto the cortical regions of the “regional mapping” macaque atlas of Kötter and Wanke (129), mirroring data between hemispheres (46).

#### Mouse brain gene expression from in situ hybridization

Mouse gene expression profiles were obtained using in situ hybridization data from the Allen Mouse Brain Atlas (44). We followed the same preprocessing as recently described (169). Briefly, the Allen Mouse Brain Atlas consists of data acquired from a pipeline that includes semi-automated riboprobe generation, tissue preparation and sectioning, in-situ hybridization (ISH), imaging, and data post-processing. These data were acquired both sagittally and (for a smaller set of genes) coronally, and were further processed, aligned by the Allen Institute to their Common Coordinate Framework version 3 (CCFv3) reference atlas (170) and summarized voxelwise through a measure termed gene expression energy (defined as the sum of expressing pixel intensity divided by the sum of all pixels in a division), resulting in 3D gene expression images at a 200 *μ*m isotropic resolution. This gene expression energy increases in regions of high expression, and is bounded by zero in regions of no expression. For our study, we used gene expression energy data from the coronal dataset (4345 gene expression images corresponding to 4082 unique genes), because of its whole-brain coverage and data quality. **(author?)** (171) provide tools to work with this gene expression data; these tools are available online (github.com/DJFernandes/ABIgeneRMINC). Voxelwise gene expression data were further summarized as normalized mean expression within regions of interest as defined by the CCFv3 reference atlas. For each ROI and each gene, voxelwise expression energy data were averaged over voxels containing valid expression signal. Finally, independently for each region, gene expression data were normalized by regressing out mean gene expression across the brain.

We also replicate our main results using mouse gene expression data from two recently released alternative databases from MERFISH, as reported in (60) and (61). Briefly, the database from (60) is a whole mouse brain spatial transcriptomics dataset containing 3.9 million segmented cells passing quality control (MERFISH-C57BL6J-638850). It was acquired using multiplexed error-robust fluorescence in situ hybridization (MERFISH) technique with a 500 gene panel. The MERFISH data was registered to the Allen CCFv3 standard template, acquired CCF coordinates for each segmented cell, and assigned into pre-defined brain structures. A separate single-cell RNA sequencing (scRNA-seq) dataset was acquired using 10Xv3 technique and imputed (projected) into MERFISH space, deriving 8,460 marker genes for each MERFISH cell. A region-by-gene matrix was derived from the cell-by-gene tabular data by aggregating within the assigned Allen CCFv3 atlas regions and taking the average hierarchically following a pre-defined simplified anatomical hierarchy. The (61) database is a whole mouse brain spatial transcriptomics dataset acquired using MERFISH technique with a 1122 gene panel, containing 9.3 million segmented cells passing quality control (Zhuang-ABCA-1/2/3/4). The MERFISH data was registered to the Allen CCFv3 standard template, and coordinates were acquired for 5.4 million cells, which were then assigned into pre-defined brain structures. We derived the region-by-gene matrix similarly as above. Out of 81 brain-related genes, 19 are available in the Yao database, and 36 are available in the Zhang database.

### A. Macaque parvalbumin density from immunohistochemistry

Burt and colleagues (62) assembled data on the mmunohistochemically measured densities of parvalbumin-expressing inhibitory interneurons for several macaque brain areas, from multiple immunohistochemistry studies (172–175). We used these data as provided by (46), who mapped parvalbumin density onto the Regional Mapping macaque atlas used in the present study.

### Computational neural mass model

Below, we describe the whole-brain computational modelling framework, as used in our previous work (119). Due to its multi-platform compatibility, low memory usage, and high speed, we used the recently developed and publicly available FastDMF library (57). The reader is referred to (57, 59) for details of the model’s implementation. Briefly, macroscale whole-brain computational models represent regional activity in terms of two key ingredients: (i) a biophysical model of each region’s local dynamics; and (ii) inter-regional anatomical connectivity. Thus, such *in silico* models provide a wellsuited tool to investigate how the structural connectivity of the brain shapes the corresponding macroscale neural dynamics (52, 53, 58, 59). In particular, the Dynamic Mean Field (DMF) model employed here simulates each region (defined via a species-specific brain parcellation scheme) as a macroscopic neural field comprising mutually coupled excitatory and inhibitory populations, providing a neurobiologically plausible account of regional neuronal firing rate. Specifically, the model simulates local biophysical dynamics of excitatory (NMDA) and inhibitory (GABA) neuronal populations, interacting over long-range neuroanatomical connections. Here, we used a consensus human connectome reconstructed from *in vivo* diffusion MRI tractography.

The following differential equations therefore govern the model’s behaviour:

(1) $$I_{n}^{\left(E\right)}=W_{E}I_{0}+w_{+}J_{NMDA}S_{n}^{\left(E\right)}+\;\;GJ_{NMDA}\sum_{P=1}^{N}C_{np}S_{p}^{\left(E\right)}-J_{n}^{FIC}S_{n}^{\left(I\right)}$$

(2) $$I_{n}^{\left(I\right)}=W_{I}I_{0}+J_{NMDA}S_{n}^{\left(E\right)}-S_{n}^{\left(I\right)}$$

(3) $$r_{n}^{\left(E\right)}=F\left(I_{n}^{\left(E\right)}\right)=\frac{g_{E}\;\left(I_{n}^{\left(E\right)}\;-\;I_{thr}^{\left(E\right)}\right)}{1\;-\;exp\;\left(-d_{E}g_{E}\;\left(I_{n}^{\left(E\right)}\;-\;I_{thr}^{\left(E\right)}\right)\right)}$$

(4) $$r_{n}^{\left(I\right)}=F\left(I_{n}^{\left(I\right)}\right)=\frac{g_{n}^{NM}g_{I}\;\left(I_{n}^{\left(I\right)}\;-\;I_{thr}^{\left(I\right)}\right)}{1\;-\;exp\;\left(-d_{I}g_{n}^{NM}g_{I}\;\left(I_{n}^{\left(I\right)}\;-\;I_{thr}^{\left(I\right)}\right)\right)}$$

(5) $$\frac{dS_{n}^{\left(E\right)}(t)}{dt}=\frac{S_{n}^{\left(E\right)}}{\tau_{NMDA}}+\left(1+S_{n}^{\left(E\right)}\right)\gamma r_{n}^{\left(E\right)}+\sigma v_{n}(t)$$

(6) $$\frac{dS_{n}^{\left(I\right)}(t)}{dt}=\frac{S_{n}^{\left(I\right)}}{\tau_{GABAA}}+r_{n}^{\left(I\right)}+\sigma v_{n}(t)$$

Following previous work (57–59, 119), for each excitatory (E) and inhibitory (I) neural mass, the quantities $I_{n}^{\left(E,I\right)},\;r_{n}^{\left(E,I\right)}$, and $S_{n}^{\left(E,I\right)}$ represent its total input current (nA), firing rate (Hz), and synaptic gating variable, respectively. The function F(⋅) is the transfer function (or F–I curve), representing the non-linear relationship between the input current and the output firing rate of a neural population. Finally, $J_{n}^{FIC}$ is the local feedback inhibitory control of region n, which is optimized to keep its average firing rate at approximately 3 Hz, and $\nu_{n}$ is uncorrelated Gaussian noise injected to region n. The model’s fixed parameters are reported in S6. The code used to run all the simulations in this study was written in optimised C++ using the high-performance library *Eigen*. The C++ core of the code, together with Python and Octave/Matlab interfaces is publicly available as FastDMF and maintained at http://www.gitlab.com/concog/fastdmf (57).

#### Human structural connectome from Human Connectome Project

We adopted previously reported procedures to reconstruct the human connectome from DWI data. The minimally-preprocessed DWI HCP data (151) were corrected for eddy current and susceptibility artifact. DWI data were then reconstructed using q-space diffeomorphic reconstruction (QSDR (176)), as implemented in DSI Studio (www.dsi-studio.labsolver.org). QSDR calculates the orientational distribution of the density of diffusing water in a standard space, to conserve the diffusible spins and preserve the continuity of fiber geometry for fiber tracking. QSDR first reconstructs diffusion-weighted images in native space and computes the quantitative anisotropy (QA) in each voxel. These QA values are used to warp the brain to a template QA volume in Montreal Neurological Institute (MNI) space using a nonlinear registration algorithm implemented in the statistical parametric mapping (SPM) software. A diffusion sampling length ratio of 2.5 was used, and the output resolution was 1 mm. A modified FACT algorithm (177) was then used to perform deterministic fiber tracking on the reconstructed data, with the following parameters (178): angular cutoff of 55 °, step size of 1.0 mm, minimum length of 10 mm, maximum length of 400 mm, spin density function smoothing of 0.0, and a QA threshold determined by DWI signal in the cerebrospinal fluid. Each of the streamlines generated was automatically screened for its termination location. A white matter mask was created by applying DSI Studio’s default anisotropy threshold (0.6 Otsu’s threshold) to the spin distribution function’s anisotropy values. The mask was used to eliminate streamlines with premature termination in the white matter region. Deterministic fiber tracking was performed until 1,000,000 streamlines were reconstructed for each individual.

For each individual, their structural connectome was reconstructed by drawing an edge between each pair of regions i and j from the Schaefer cortical atlas (149) if there were white matter tracts connecting the corresponding brain regions end-to-end; edge weights were quantified as the number of streamlines connecting each pair of regions, normalised by ROI distance and size.

A group-consensus matrix *A* across participants was then obtained using the distance-dependent procedure of Betzel and colleagues, to mitigate concerns about inconsistencies in reconstruction of individual participants’ structural connectomes (179). This approach seeks to preserve both the edge density and the prevalence and length distribution of inter- and intra-hemispheric edge length distribution of individual participants’ connectomes, and it is designed to produce a representative connectome (179, 180). This procedure produces a binary consensus network indicating which edges to preserve. The final edge density was 27%. The weight of each non-zero edge is then computed as the mean of the corresponding non-zero edges across participants.

#### Model fitting

Following (57, 59), all but one of the DMF model parameters are set as per (181), leaving only one free parameter, known as “global coupling” and denoted by G, which controls the overall strength of signal transmission between brain regions (conductivity of the white matter fibers is assumed to be constant across the brain). We first tune G to match the high-quality Human Connectome Project functional MRI dataset (150). We set the model to have the same TR as the HCP fMRI data (0.72s), with data filtered in the same frequency band (0.0080–09 Hz). Then we use a Bayesian optimiser to identify the G value that maximises the fit between model and empirical HCP fMRI data (57). Goodness of fit was quantified as the similarity between the data’s and the model’s functional connectivity dynamics (FCD), computed as follows. First, we obtained Pearson correlation matrices between regional fMRI signal time-series, computed within a sliding window of 30 TRs with increments of 3 TRs (59, 119). Subsequently, the resulting matrices of functional connectivity at times $t_{i}$ and $t_{j}$ were themselves correlated, for each pair of timepoints $t_{i}$ and $t_{j}$, thereby obtaining an FCD matrix of time-versus-time correlations. Thus, each entry in the FCD matrix represents the similarity between functional connectivity patterns at different points in time. The best-fitting value of the G parameter is identified as the one that minimises the Kolmogorov-Smirnov distance between the histograms of empirical (group-wise) and simulated FCD values (obtained from the upper triangular FCD matrix) (59, 119). Use of the FCD is well established as fitting target for the DMF model (57, 59, 119), and we note that this measure is not one of the measures that we investigate in the present study, thereby avoiding the issue of circularity.

Then, starting from this independently parameterised model (obtained for G=1.8), we systematically vary the G parameter from 1.4 to 2.1, in increments of 0.1. At each value of G we simulate regional BOLD signals for a sample of N=40 simulations. For each region in each simulation at each value of G, we then extract the *catch22* representative reduced set of dynamical features from (47). Out of this set dynamical features, 13 exhibit consistent changes across species as a result of anaesthesia (i.e., they either consistently increase across species during anaesthesia compared to wakefulness, or consistently decrease). For each value of G, we ask how many among these 13 dynamical features exhibit a change (increase or decrease from the tuned model) in the same direction as the direction of consistent change observed under anaesthesia across species. This procedure allows us to find the value of G that most appropriately reproduces the empirical dynamical signature of anaesthesia. At each value fo G we also compute the dynamical profile similarity and functional connectivity for each simulation, to assess how the correlation between the two changes as a function of the global inter-regional coupling.

### Statistical analyses

#### Anaesthetic effect size

For each contrast (awake vs anaesthesia) in each dataset, we consider each region/neuron and feature in turn. We use the measures of effect size toolbox for MATLAB https://github.com/hhentschke/measures-of-effect-size-toolbox (182) to estimate an effect size for the difference between that feature’s values during wakefulness and during anaesthesia using Hedge’s measure of the standardized mean difference, g, which is interpreted in the same way as Cohen’s d, but more appropriate for small sample sizes (36). Note that features were not normalised across regions before analysis, as doing so could obscure differences in magnitude. Although hctsa features span several orders of magnitude, such that raw differences cannot be meaningfully compared across features, effect sizes are expressed in units of standard deviation, and therefore they are readily comparable across features and also across regions and datasets. For each contrast in each dataset, this procedure produces a matrix of awake-vs-anaesthesia effect sizes, with dimensions n regions/neurons × f features (with f=6958 for all species after post-filtering).

#### Identification of consistently-perturbed features

To identify dynamical features that are consistently altered across species and across anaesthetics, we perform two steps. The first step is performed within-species. For each species, we compute the mean feature-wise effect sizes across all contrasts belonging to that same species, and across all regions/neurons. For each species, this step produces a vector of 6958 features, where each entry represents the mean effect size that anaesthesia induces in that features, on average across regions and anaesthetics (if applicable). The second step is performed across species. For each feature, we ask whether its direction of change (regardless of magnitude) is the same in every species (all positive or all negative). We discard features that do not meet this criterion. The outcome of this procedure is a set of dynamical features that are consistently altered in the same direction across regions/neurons, across anaesthetics, and across species.

#### Feature category enrichment

The hctsa features are not evenly distributed among the 18 broad categories of dynamics. Simply counting the relative prevalence of each category among a subset of features of interest (such as the subset of features that are consistently altered by anaesthesia) is therefore not appropriate. Rather, we should ask whether our subset of interest is enriched for any specific feature category, such that the prevalence of that category is greater in the subset, than we would expect to occur just by chance in a subset of equal size.

To implement this test, we first compute the empirical prevalence of each feature category in our subset of n features of interest. We then randomly sample *n* features (without replacement) and count how many features belong to each category. We repeat this procedure 10 000 times to obtain a null distribution of category frequencies for a subset of size n. Finally, we compute a z-score for each empirically observed category frequency, against this null distribution. A positive z-score indicates that a feature category is observed more often among the n features of interest, than would be expected by chance. Note that more sophisticated tests could also be devised. For example, features are not independent and may exhibit complex relationships both within and across categories. The same pair of features could conceivably exhibit different levels of similarity in different datasets, which will influence their probability of co-occurring in a subset of interest.

#### Multivariate association with Partial Least Squares

Partial Least Squares (PLS) analysis was used to relate regional gene expression to anaesthetic-induced changes in local dynamics in a multivariate fashion. PLS analysis is an unsupervised multivariate statistical technique that decomposes relationships between two datasets $X_{n\times g}$ and $Y_{n\times f}$ into orthogonal sets of latent variables with maximum covariance, which are linear combinations of the original data (48, 49). In the present case, $X_{n\times g}$ is regional gene expression across n regions (100 for the human; 82 for the macaque; and 72 for the mouse) and g genes (the same set of 81 for each species). $Y_{n\times f}$ is the matrix of regional anaesthetic-induced changes in dynamical features, across f features.

To ensure consistent gene-dynamics relationships across species, we perform this analysis on matrices $X_{n\times g}$ and $Y_{n\times f}$ obtained by vertically concatenating the species-specific matrices for human, macaque, and mouse. Within each species, we separately z-score the $X_{n\times g}$ and $Y_{n\times f}$ matrices column-wise. We then concatenate vertically the species-specific X matrices of z-scores, and we likewise concatenate vertically the species-specific Y matrices of z-scores. This is possible because the columns are the same for each across all three species: the same 81 genes, and the same dynamical features. This results in a single $X_{n\times g}$ matrix and a single $Y_{n\times f}$ matrix, each with n=100+82+72=254 rows.

PLS finds components from the predictor variables (regional gene expression) that have maximum covariance with the response variables (regional changes in dynamical features). The PLS components (i.e., linear combinations of the weighted variables) are ranked by the covariance between predictor and response variables so that the first few PLS components provide a low-dimensional representation of the covariance between the higher-dimensional data matrices. Concretely, this is achieved by performing singular value decomposition (SVD) on the matrix $Y^{\prime }X$, such that:

(7) $$Y^{\prime }X=U_{g\times t}S_{t\times t}V_{t\times t}^{\prime },$$

where $U_{g\times t}$ and $V_{t\times t}$ are orthonormal matrices consisting of left and right singular vectors, and $S_{t\times t}$ is a diagonal matrix of singular values. The *i*th columns of U and V constitute a latent variable, and the *i*th singular value in S represents the covariance between singular vectors. The *i*th singular value is proportional to the amount of covariance between gene expression and anaesthetic-induced dynamical changes captured by the *i*th latent variable, where the effect size can be estimated as the ratio of the squared singular value to the sum of all squared singular values.

For our main analysis, we use the subset of *catch22* representative features (excluding *acf_timescale* which did not return finite values for all regions; and *ami2* which did not pass our pre-filtering step, leaving the same 20 dynamical features available for use in all species). As a further validation, we also repeat the analysis using all 541 consistent features. Significance of the latent variables is assessed against a null distribution of null maps with preserved spatial autocorrelation, as described below.

#### Null model

The statistical significance of the covariance explained by each PLS model is tested by permuting the response variables 10 000 times while considering the spatial dependency of the data by using spatial autocorrelation-preserving permutation tests, to control for the spatial autocorrelation inherent in neuroimaging data, which can induce an inflated rate of false positives (50, 183).

For each species, we generate null maps using Moran spectral randomisation based on on the inverse Euclidean distances between parcel centroids for that species, as implemented in the BrainSpace toolbox (https://brainspace.readthedocs.io/en/latest/) (51). Moran spectral randomisation quantifies the spatial autocorrelation in the data in terms of Moran’s *I* coefficient, by computing spatial eigenvectors known as Moran eigenvector maps. The Moran eigenvectors are then used to generate null maps by imposing the spatial structure of the empirical data on randomised surrogate data (51, 183). As for the empirical data, each null map is z-scored within-species and the corresponding null maps are then concatenated vertically across species, to obtain a null Y matrix. PLS analysis is then applied to the original X matrix and this null Y matrix. This procedure is repeated 10 000 times, to obtain a null distribution of the singular values associated with each latent variable. This test embodies the null hypothesis that regional gene expression and regional dynamical changes induced by anaesthesia are spatially correlated with each other only because of inherent spatial autocorrelation. The p-value is computed as the proportion of null singular values that are greater in magnitude than the empirical singular values. Thus, these p-values represent the probability that the observed spatial correspondence between genes and dynamical features could occur by randomly correlating maps with comparable spatial autocorrelation.

## Supplementary Material

Supplement 1

## Data Availability

The original pharmacological fMRI data are available from the corresponding authors of the original publications referenced herein. Human gene expression data from the Allen Human Brain Atlas (42) are available at https://human.brain-map.org/. Macaque cortical gene expression data from (43) are available at https://macaque.digital-brain.cn/spatial-omics. The dataset is provided by Brain Science Data Center, Chinese Academy of Sciences (https://braindatacenter.cn/). Mouse gene expression data from the Allen Mouse Brain Atlas (44) are available at https://mouse.brain-map.org/. The Highly Comparative Time-Series Analysis (hctsa) toolbox is freely available at https://github.com/benfulcher/hctsa. The abagen toolbox for processing of the AHBA human transcriptomic dataset is available at https://abagen.readthedocs.io/. The BrainSpace toolbox for Moran spectral randomisation is available at https://brainspace.readthedocs.io/en/latest/. The FastDMF code for whole-brain modelling is available at https://www.gitlab.com/concog/fastdmf.
