# Supplementary material for: Comprehensive profiling of anaesthetised brain dynamics across phylogeny
Source: bioRxiv. 2025 Mar 24:2025.03.22.644729. Preprint. [Version 1] doi: 10.1101/2025.03.22.644729 (PMC11974681; doi:10.1101/2025.03.22.644729)
Supplement: Supplement 1 [file media-1.pdf]

Supplementary Figures

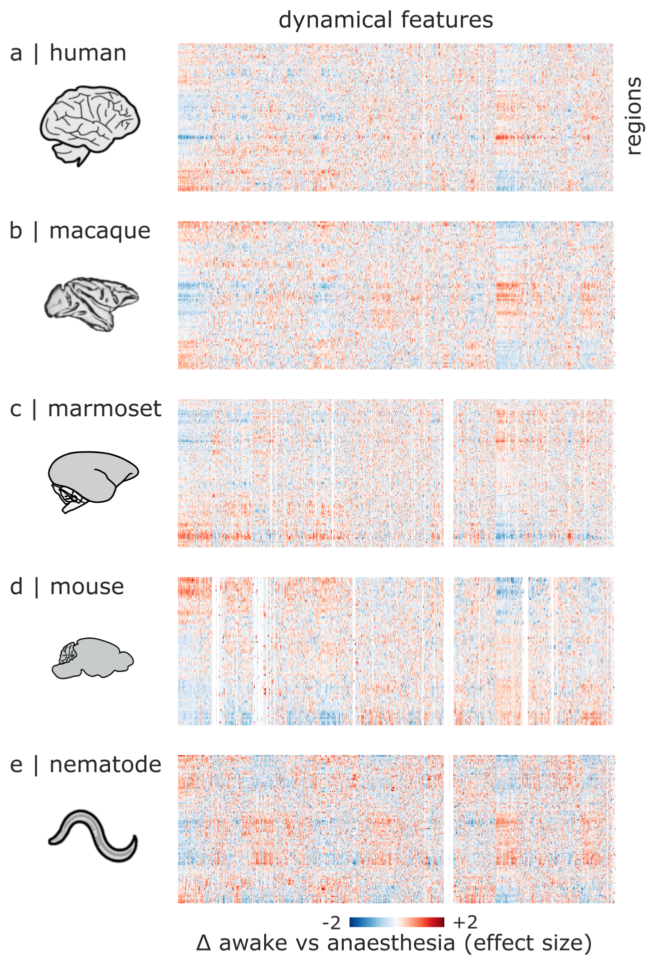

Figure S1. **Z-scored anaesthetic-induced changes in neural dynamics across species |** (**a**) Human: z-scored mean effect sizes across awake vs vol 3% sevoflurane, and recovery vs vol 3% sevoflurane. (**b**) Macaque: z-scored mean effect sizes across awake vs sevoflurane; awake vs propofol; awake vs ketamine (for the Multi-anaesthesia dataset); awake vs propofol (no DBS); CT DBS versus propofol; and CT DBS vs VT DBS. (**c**) Marmoset: z-scored mean effect sizes across awake vs sevoflurane; awake vs propofol; and awake vs isoflurane. (**d**) Mouse: z-scored mean effect sizes across awake vs halothane; and awake vs medetomidine-isoflurane. (**d**) Nematode: z-scored effect size for awake vs isoflurane. Z-scoring is performed separately for each column (feature) in each species. Rows are then reordered to highlight consistent patterns of regional variation. Features are provided in the same order across species.

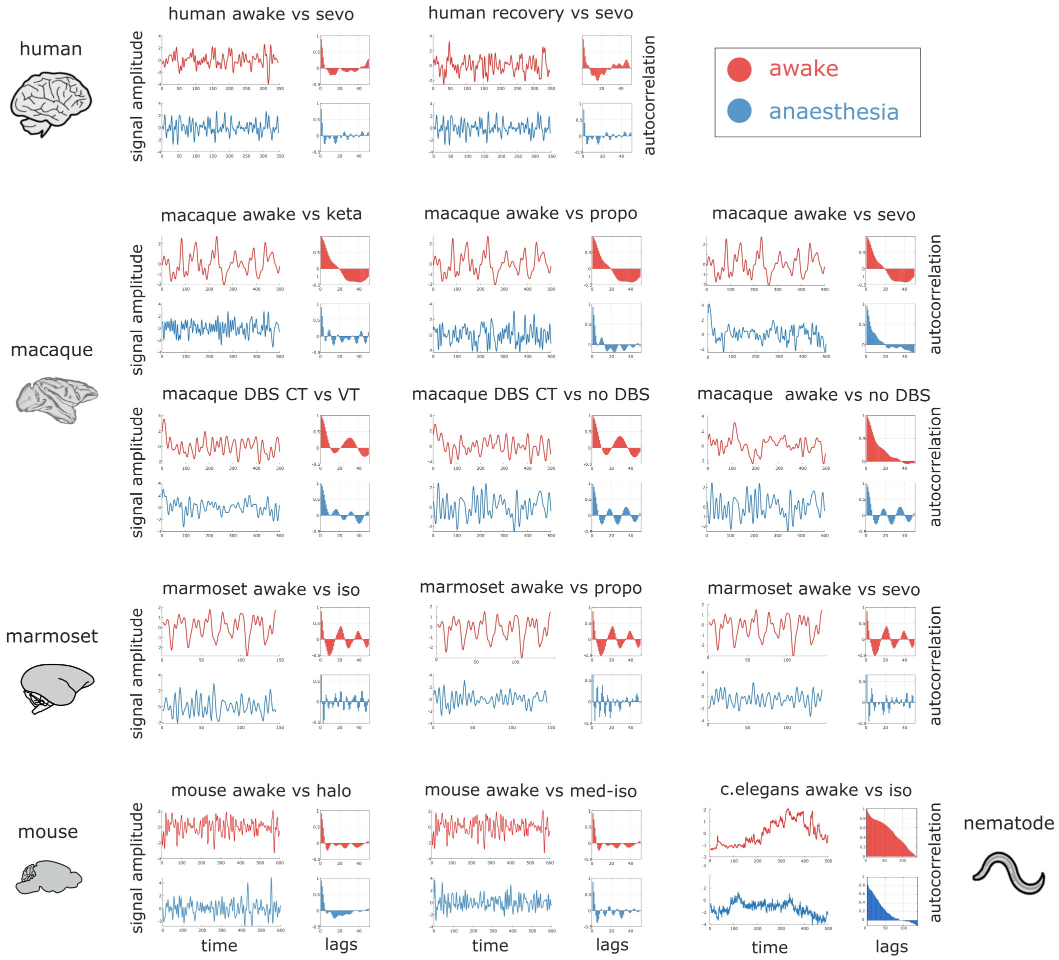

Figure S2. **Examples of anaesthetic effect on neural time-series across species |** Example time-series and their autocorrelation function (up to lag-50) during wakefulness/recovery (top) and anaesthesia (bottom), for each contrast. Red and blue indicate wakefulness and anaesthesia, respectively.

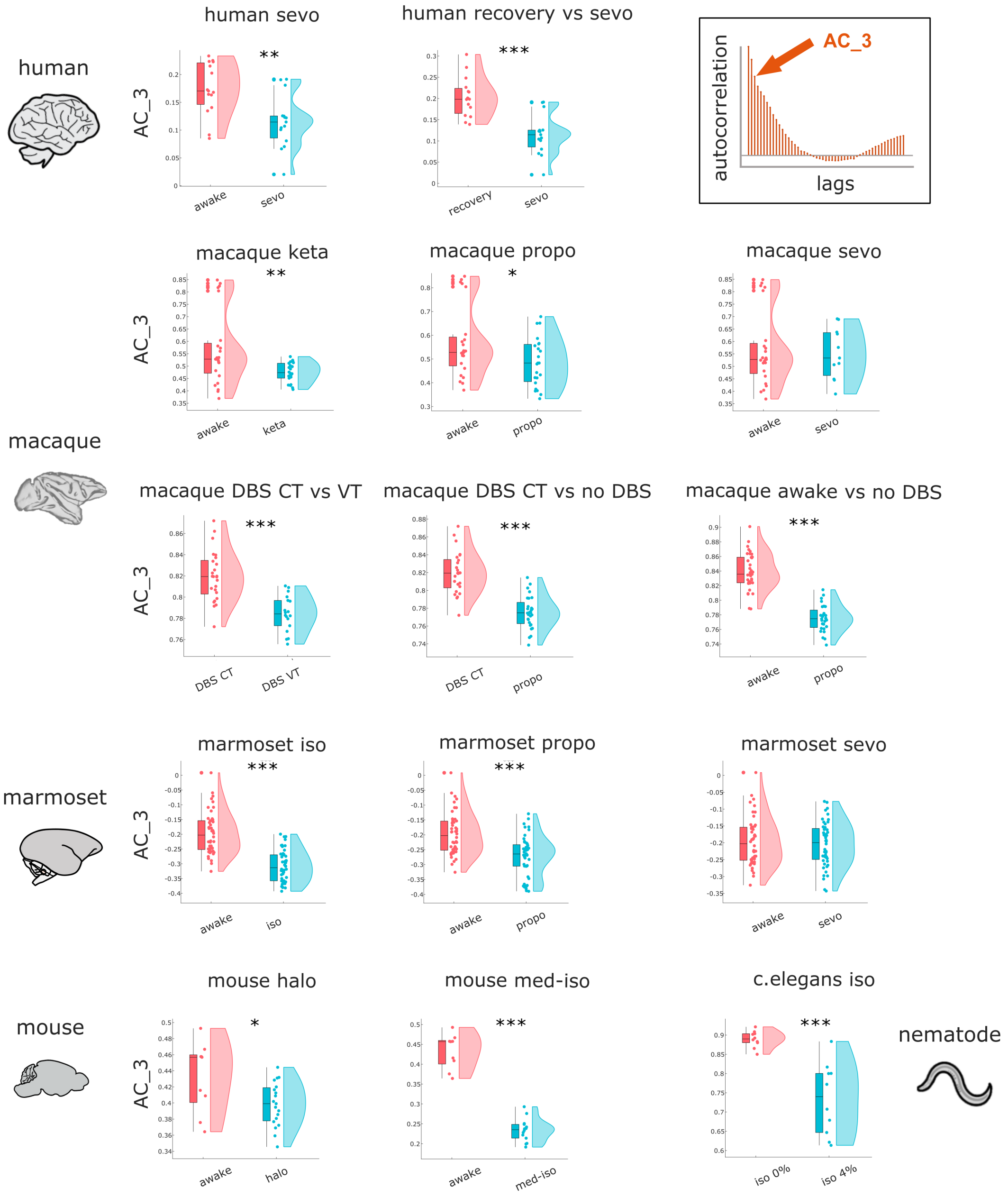

Figure S3. **Individual-level anaesthetic effect on lag-3 autocorrelation |** Ordinate represents the brain's mean value of lag-3 autocorrelation, across all regions. Each data-point represents one scan. Box-plots: center line, median; box limits, upper and lower quartiles; whiskers, 1.5× interquartile range. **, $p < 0.01$; ***, $p < 0.001$, from non-parametric permutation-based t-test (dependent samples for human and marmoset; independent samples for macaque, mouse and nematode).

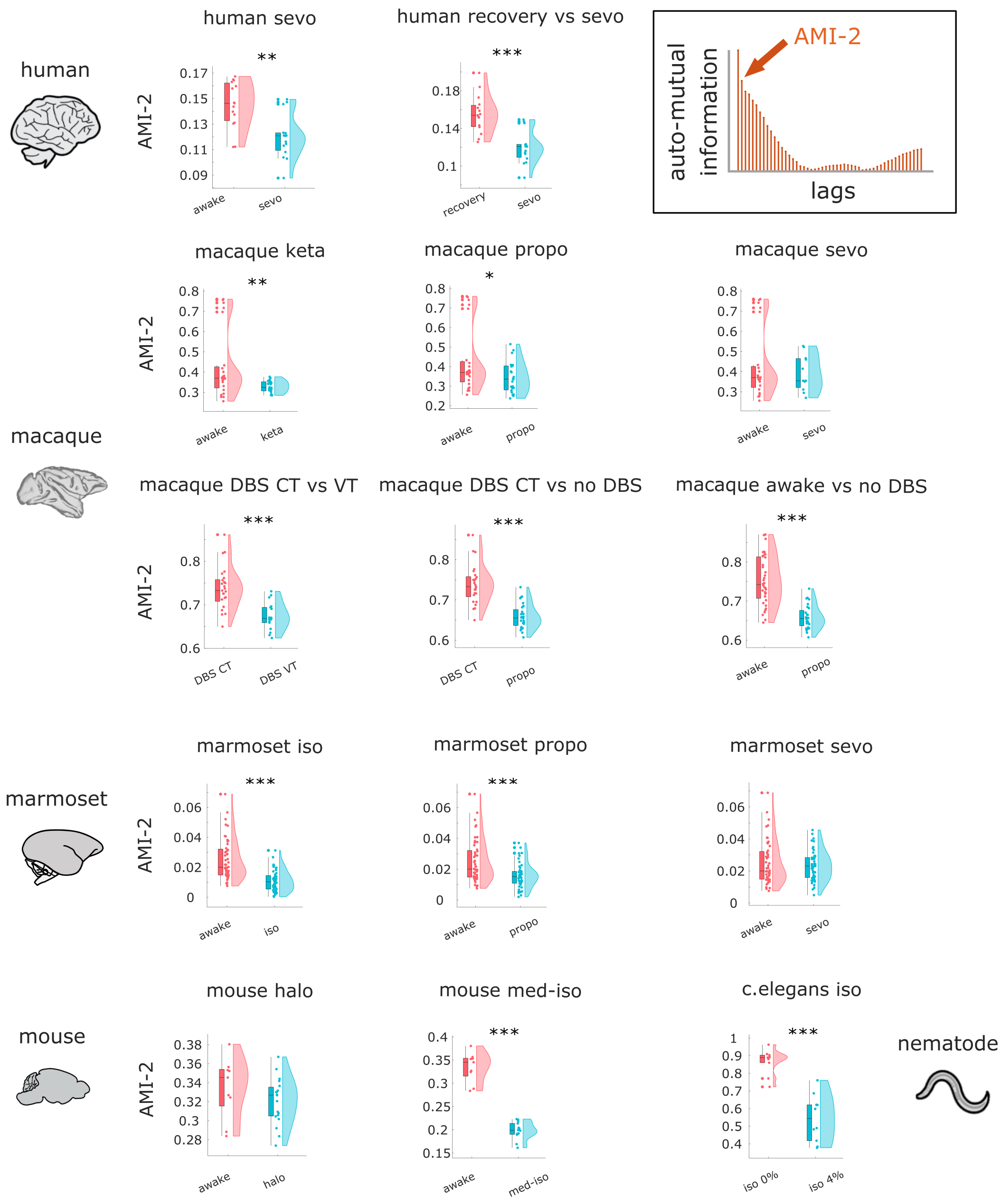

Figure S4. **Individual-level anaesthetic effect on lag-2 automutual information |** Ordinate represents the brain's mean value of lag-2 automutual information, across all regions. Each data-point represents one scan. Box-plots: center line, median; box limits, upper and lower quartiles; whiskers, $1.5\times$ interquartile range. **, $p < 0.01$; ***, $p < 0.001$, from non-parametric permutation-based t-test (dependent samples for human and marmoset; independent samples for macaque, mouse and nematode).

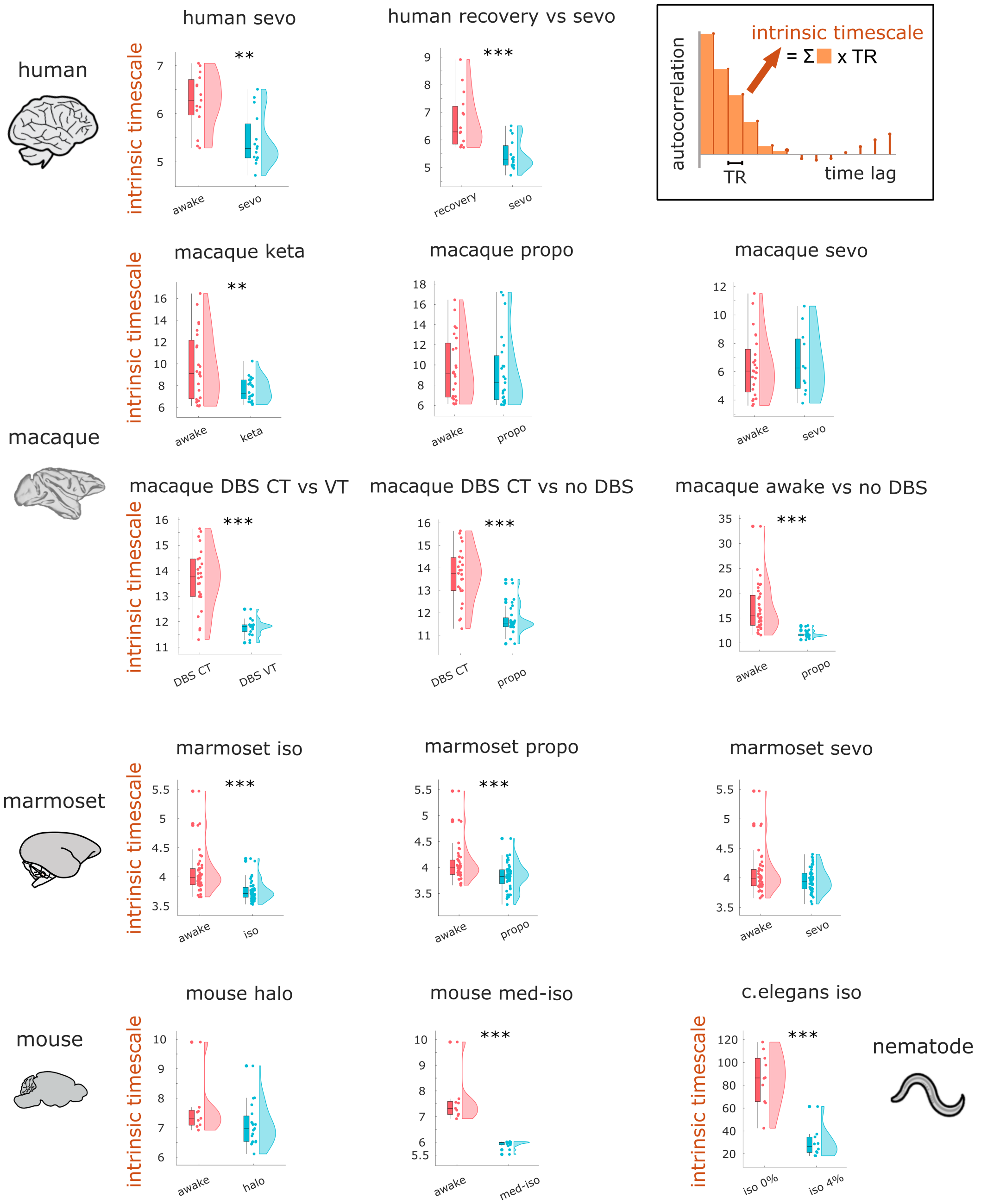

Figure S5. **Individual-level anaesthetic effect on brain-wide intrinsic neural timescales across species |** Ordinate represents the brain's mean value of intrinsic neural timescale, across all regions. Each data-point represents one scan. Box-plots: center line, median; box limits, upper and lower quartiles; whiskers, 1.5× interquartile range. **, $p < 0.01$; ***, $p < 0.001$, from non-parametric permutation-based t-test (dependent samples for human and marmoset; independent samples for macaque, mouse and nematode).

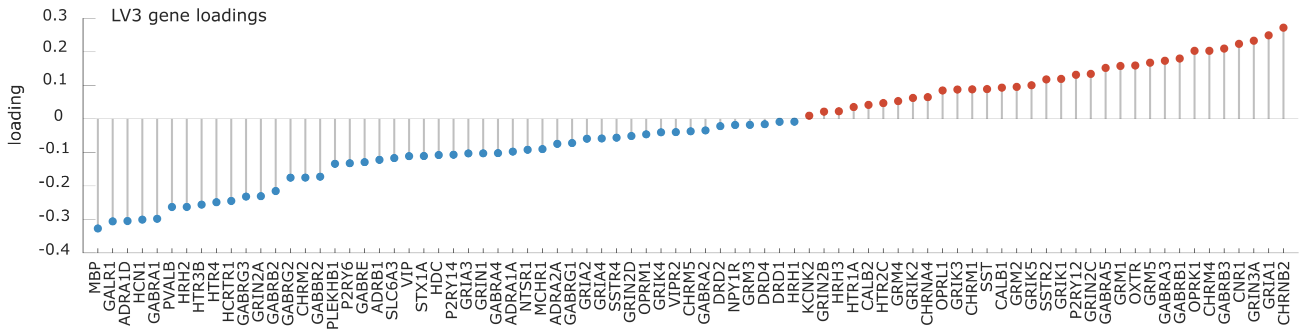

Figure S6. **Loading of each gene onto the significant dimension of multivariate association with anaesthetic-induced changes in local dynamical features |** Red indicates positive loading, blue indicates negative loading.

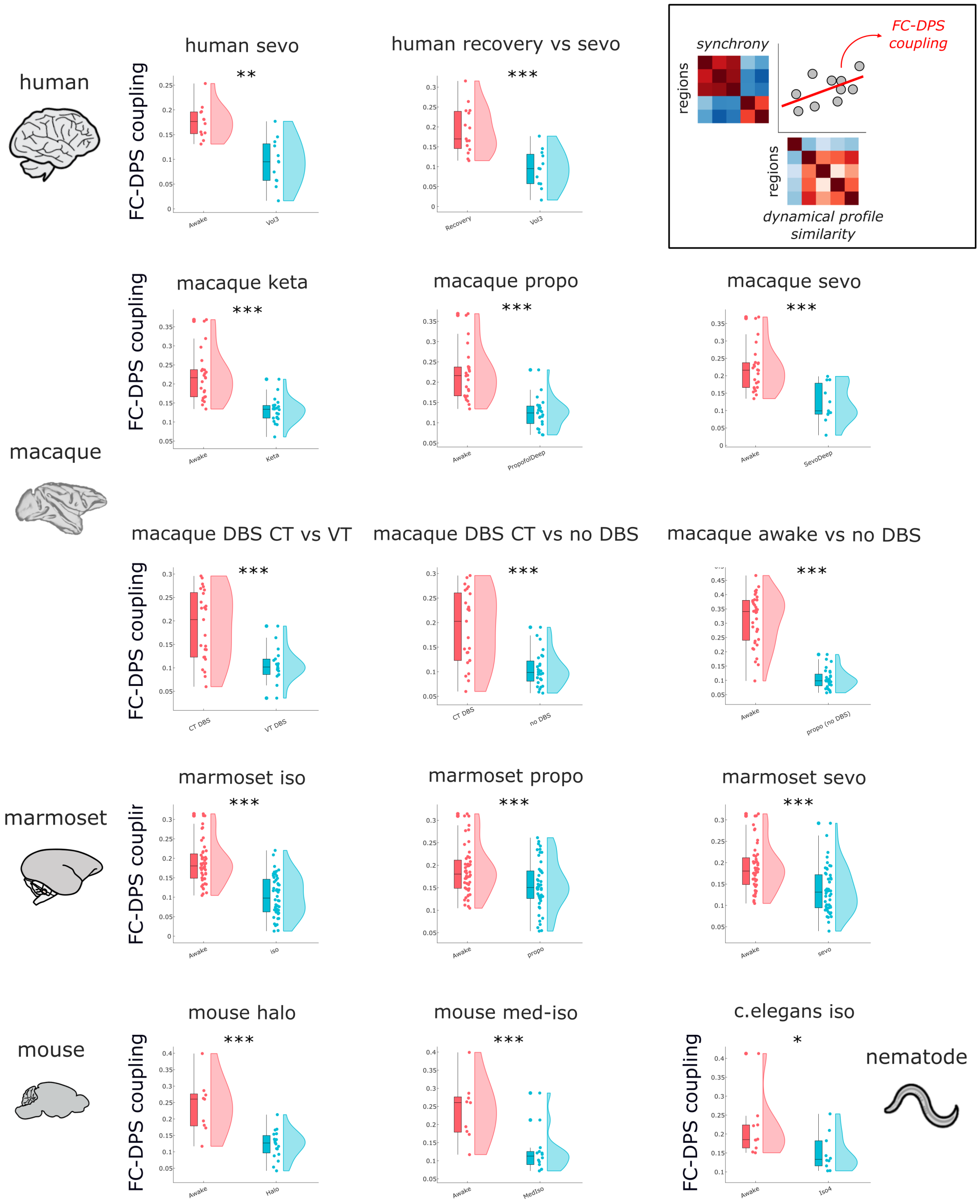

Figure S7. **Anaesthetic effect on the coupling between synchrony and dynamical profile similarity |** Ordinate represents the correlation between inter-regional synchrony and dynamical profile similarity. Each data-point represents one scan. Box-plots: center line, median; box limits, upper and lower quartiles; whiskers, $1.5\times$ interquartile range. **, $p < 0.01$; ***, $p < 0.001$, from non-parametric permutation-based t-test (dependent samples for human and marmoset; independent samples for macaque, mouse and nematode).

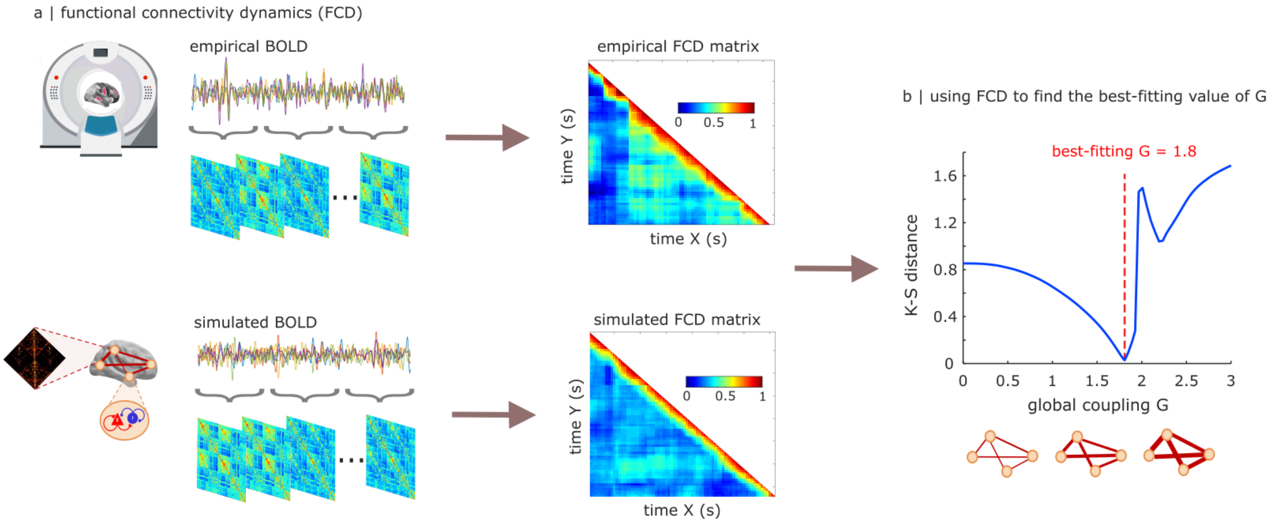

Figure S8. **Finding the optimal value of the dynamic mean-field model's global coupling parameter |** (**a**) For both empirical and simulated BOLD signals, a time-versus-time matrix of functional connectivity dynamics (FCD) is computed by correlating the time-dependent FC matrices centred at each timepoint. (**b**) Across values of the global coupling parameter *G*, we compute the KS-distance between the empirical and simulated FCD. A Bayesian optimiser (57) is used to sample different values of *G* and identify the point where the KS distance between empirical and simulated FCD is minimised, representing the point of best fit. The resulting value (here: $G = 1.8$) is used to scale the structural connectome for generating simulated time-series for the tuned model. Abscissa: *G* values. Ordinate: KS distance (lower is better).

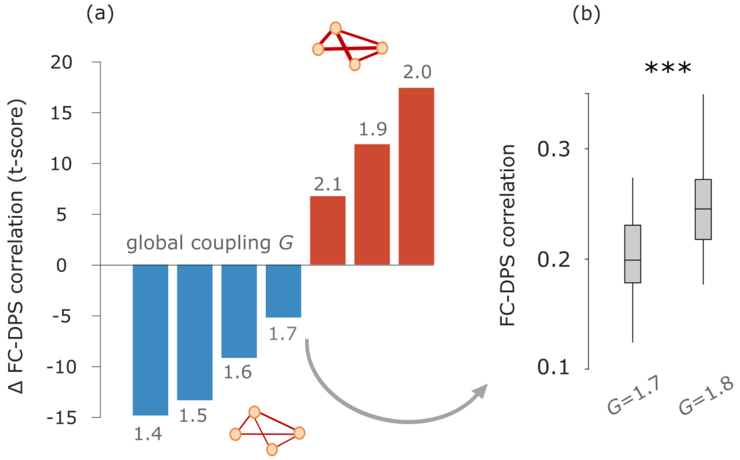

Figure S9. **Computational model with weakened inter-regional structural coupling exhibits decoupling between synchrony and dynamical profile similarity |** (**a**) Change in synchrony-dynamics correlation as a function of the global coupling *G*, against the independently parameterised model obtained at $G = 1.8$. (**b**) At $G = 1.7$, the synchrony-DPS coupling is significantly lower than at $G = 1.8$, consistent with what is observed under anaesthesia. Box-plots: center line, median; box limits, upper and lower quartiles; whiskers, $1.5\times$ interquartile range. ***, $p < 0.001$.

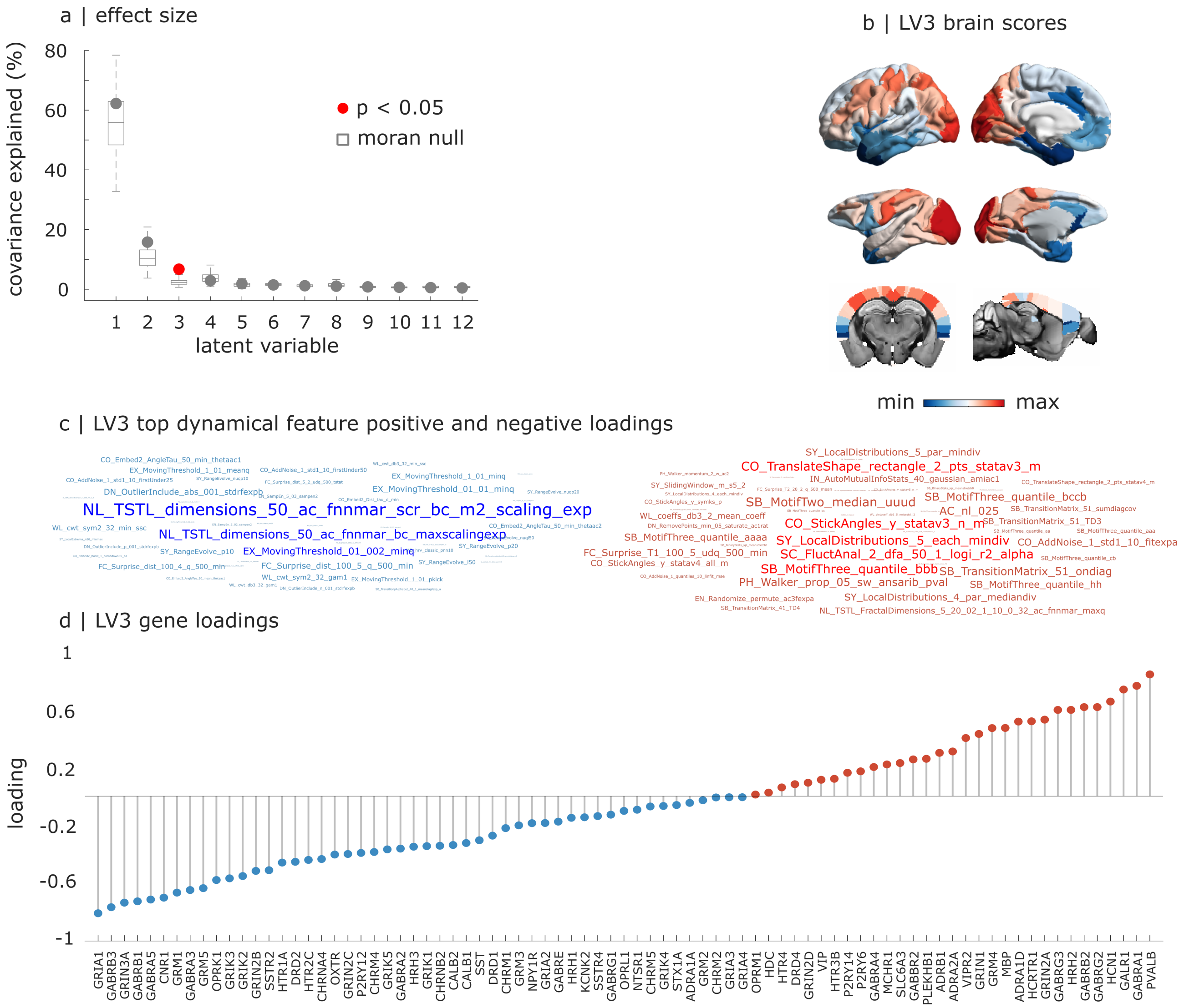

Figure S10. **Gene-dynamics association across all dynamical features that are consistently perturbed by anaesthesia |** (**a**) We find a statistically significant latent dimension of multivariate association between gene expression and anaesthetic-induced feature change (LV3). The first two latent dimensions are not significant beyond the effect of spatial autocorrelation. (**b**) Representation of the significant LV3 on the cortex of each species, delineating a phylogenetically conserved anterior-posterior gradient. (**c**) Word-clouds of features whose regional change under anaesthesia drives the association with anaesthetic-induced changes across species. Red indicates positive loading, blue indicates negative loading. (**d**) Loading of each gene onto the significant dimension of multivariate association with anaesthetic-induced changes in local dynamical features. Red indicates positive loading, blue indicates negative loading.

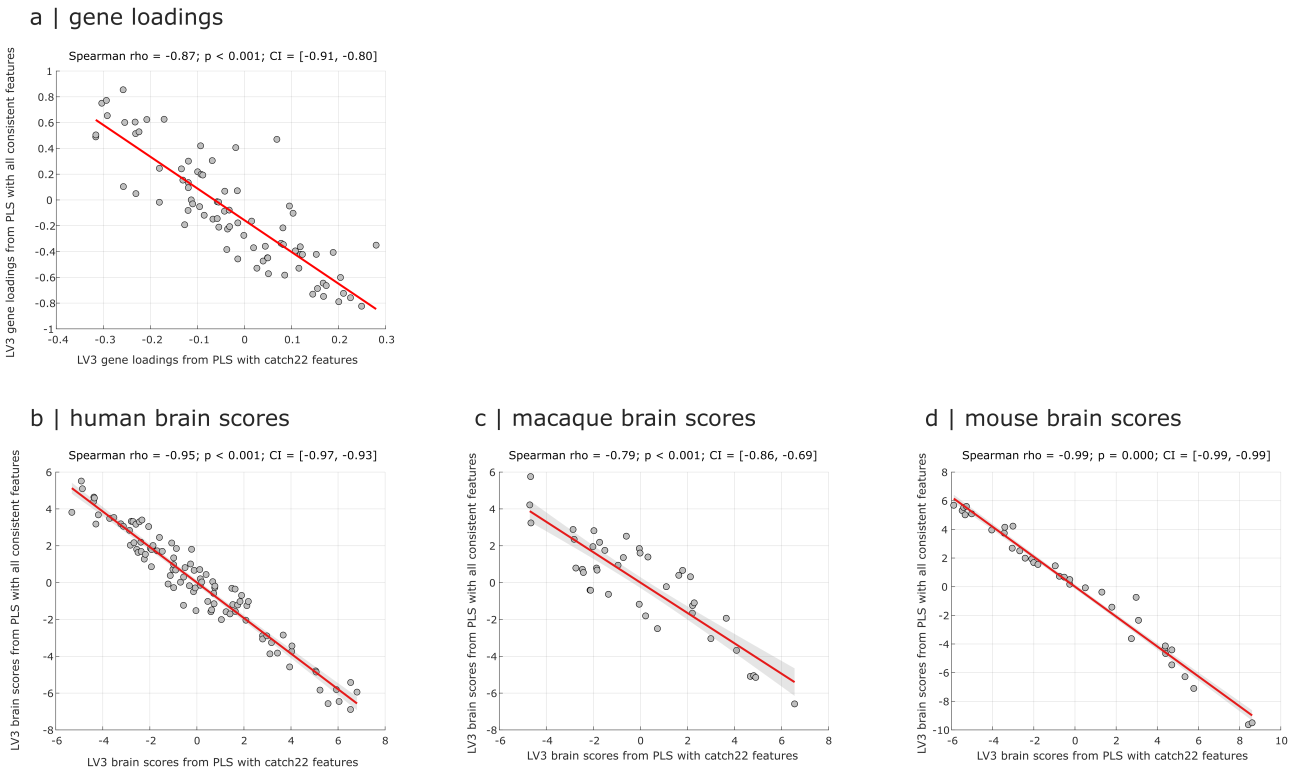

Figure S11. **Replicating phylogenetically conserved dimensions of gene-dynamics association with all dynamical features that are consistently perturbed by anaesthesia |** (**a**) There is a significant negative correlation between LV3 gene loadings obtained with the `catch22` subset of dynamical features, and obtained with the full set of 541 consistent features from `hctsa`. Note that sign is arbitrary with PLS so what matters is only the magnitude of correlation. (**b-d**) Significant negative correlations between LV3 brain scores obtained with the `catch22` subset of dynamical features (Fig. 5c), and with the full set of 541 consistent features from `hctsa` (Fig. S10b), for human (b), macaque (c) and mouse (d). Note that sign is arbitrary with PLS so what matters is only the magnitude of correlation.

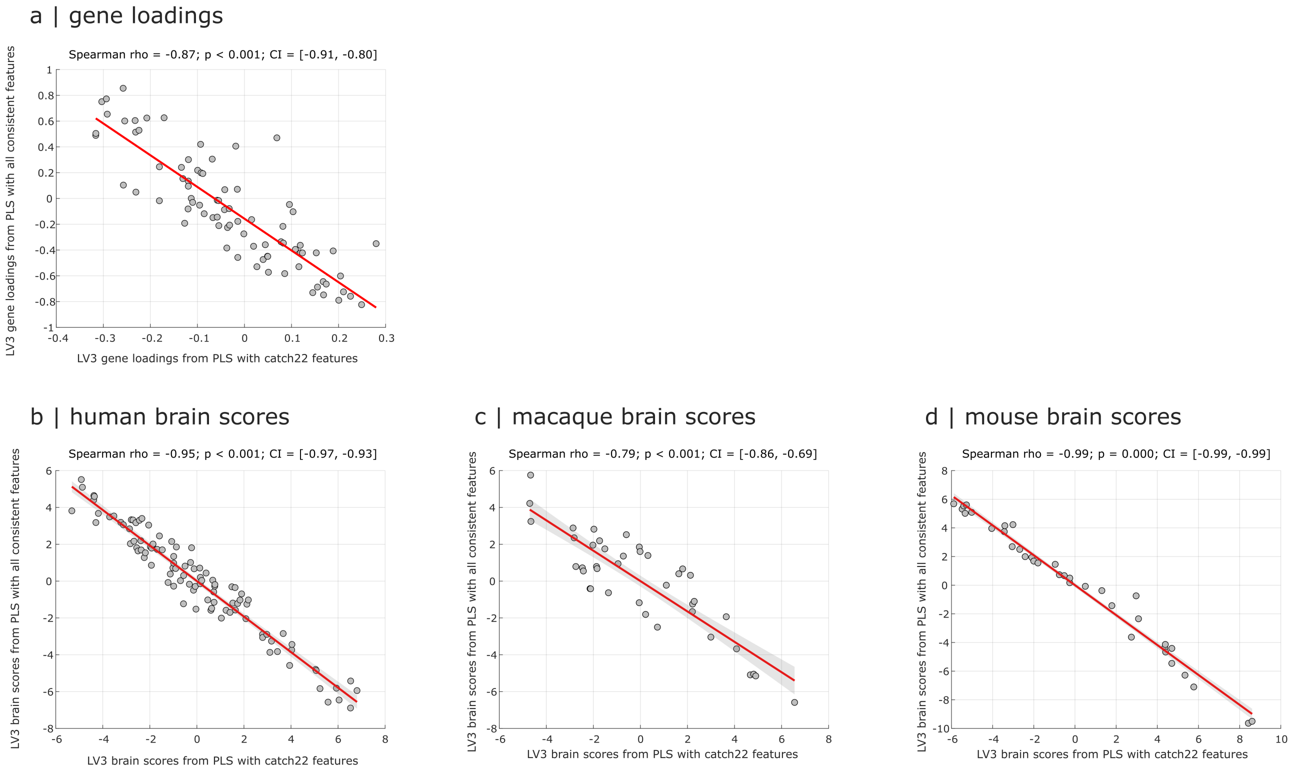

Figure S12. **Replicating phylogenetically conserved dimensions of gene-dynamics association with additional contrasts |** We repeat our PLS analysis after including additional contrasts for the human sevoflurane dataset (2% vol and burst-suppression), showing that results remain consistent, with correlated LV3 gene loadings (a), correlated LV3 dynamical feature loadings (b), and correlated LV3 brain scores for human, macaque, and mouse (c-e).

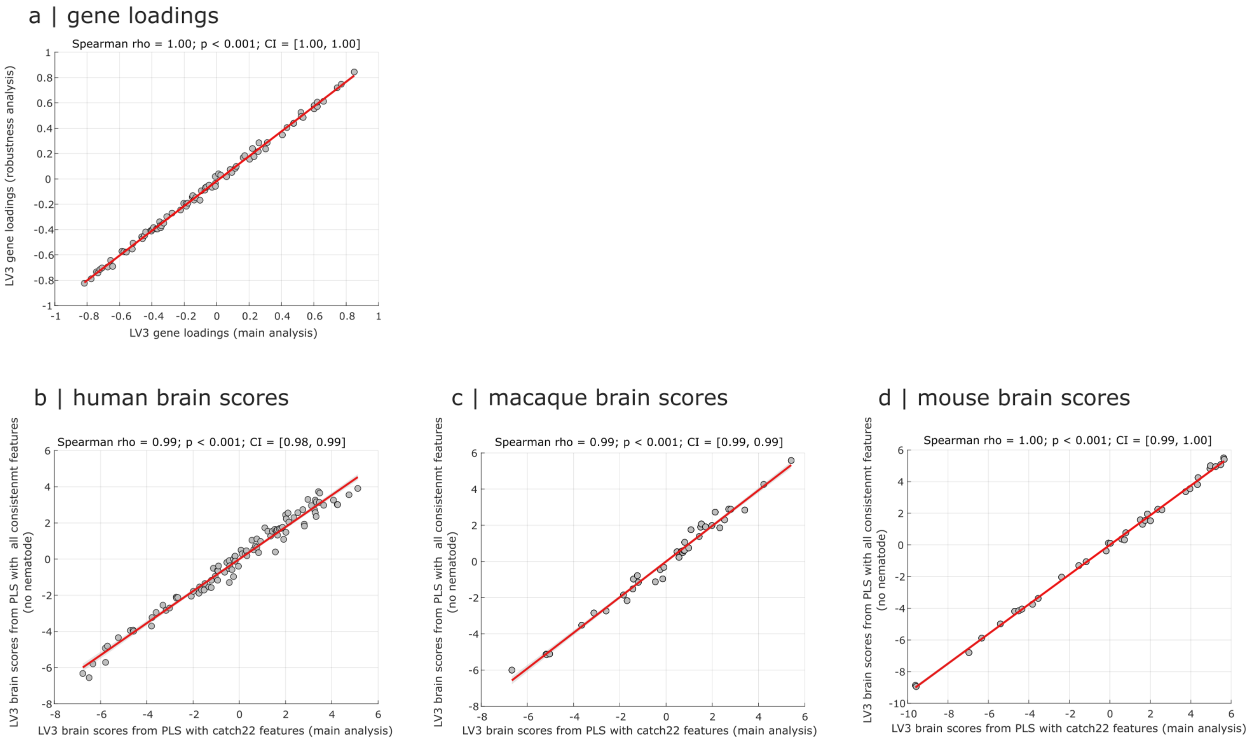

Figure S13. **Replicating phylogenetically conserved dimensions of gene-dynamics association when excluding nematode data from the definition of consistent features |** We repeat our PLS analysis after excluding the nematode data, showing that results remain consistent, with correlated LV3 gene loadings (a), and correlated LV3 brain scores for human, macaque, and mouse (b-d).

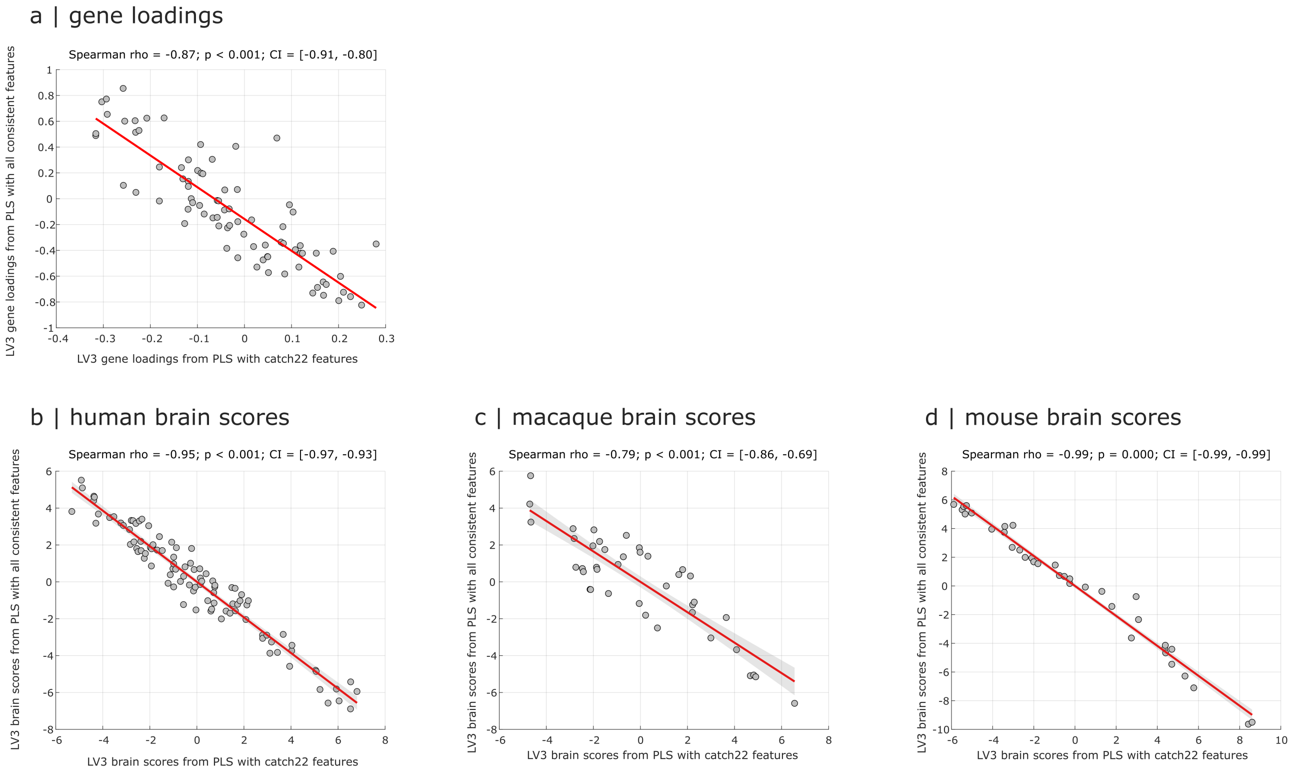

Figure S14. **Replicating human PLS brain scores with RNA-seq gene expression |** Abscissa: human LV3 brain scores from microarray gene expression. Ordinate: human LV3 brain scores from RNA-seq gene expression. Each data-point is one cortical region.

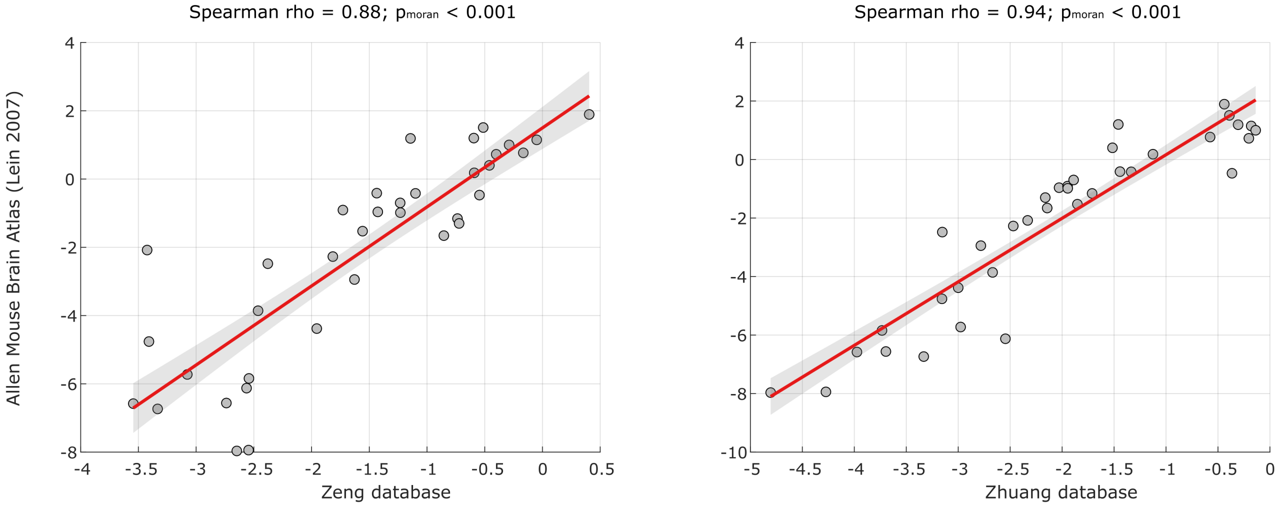

Figure S15. **Replicating mouse PLS brain scores with alternative databases of mouse gene expression |** Left: replication with the database of (60). Right: replication with the database of (61). Axes reflect LV3 brain scores for the muse. Each data-point is one cortical region.

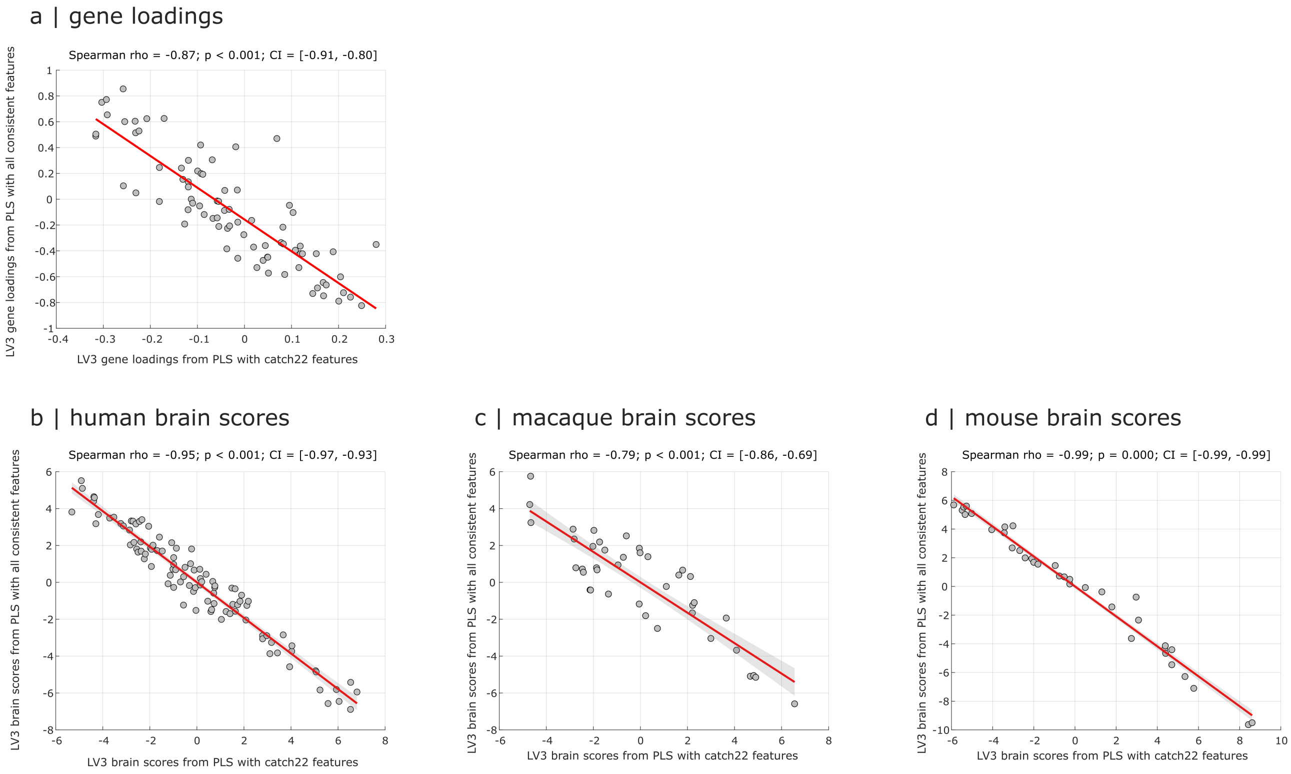

Figure S16. **Macaque PLS brain scores are significantly associated with regional parvalbumin protein density |** Abscissa: macaque LV3 brain scores. Ordinate: macaque regional parvalbumin protein density from immunohistochemistry (62). Each datapoint is one cortical region.

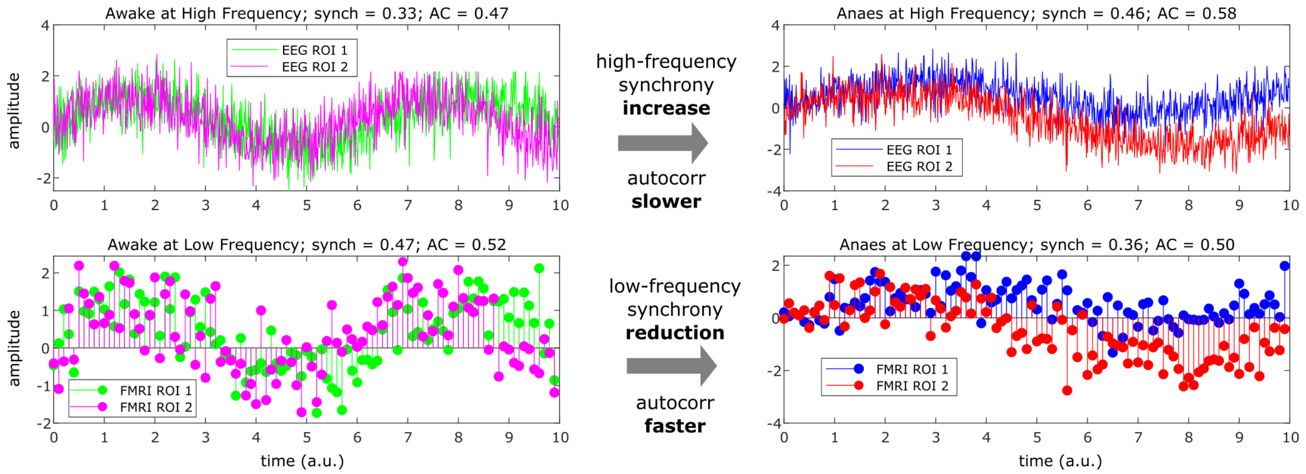

Figure S17. **Toy example showing that sampling rate influences estimated changes in synchrony and autocorrelation in the same time-series |** Left: the same pair of synthetic time-series are shown, sampled at fast rate (top) or slow rate (100x slower; bottom). Right: a different pair of synthetic time-series, also sampled at fast or slow rate. When considering the fast (EEG-like) sampling, the time-series on the right appear more synchronous and more autocorrelated than those on the left, coinciding with the effects of anaesthesia on EEG signals described in the literature. However, when considering the slower (fMRI-like) sampling, the time-series on the right appear *less* synchronous and *less* autocorrelated than those on the left, matching the results from the present report. This toy example shows that observing lower autocorrelation and reduced synchrony at slow timescales is compatible with higher autocorrlation and more synchrony at fast timescales, not only in theory but also in practice.

Supplementary Tables

Species	Awake conditions	Anaesthesia conditions	Imaging modality	Temporal resolution	Time points	Spatial coverage	Reference
Human (Homo sapiens) N=15	-awake (15 scans)<br>-recovered (15 scans)	sevoflurane (15 scans)	fMRI 3T BOLD	1838 ms	346	100	Ranft et al., (2016) Anesthesiology
Macaque (Macaca mulatta) N=5	awake (24 scans)	-sevoflurane (11 scans)<br>-propofol (23 scans)<br>-ketamine (22 scans)	fMRI 3T MION	2400 ms	500	82	Uhrig et al., (2018) Anesthesiology
Macaque (Macaca mulatta) N=5	-awake (36 scans)<br>-reawakened by central thalmus DBS (25 scans)	-propofol (28 scans)<br>-propofol with ventral thalamus DBS (18 scans)	fMRI 3T BOLD	1250 ms	500	82	Tasserie et al., (2022) Science Advances
Marmoset (Callithrix jacchus) N=4	awake (48 scans)	-propofol (48 scans)<br>-sevoflurane (48 scans)<br>-isoflurane (48 scans)	fMRI 9.4T BOLD	2000 ms	150	104	Muta et al., (2023) Cerebral Cortex
Mouse (Mus musculus) N=43	awake (10 scans)	-med-iso (14 scans)<br>-halothane (19 scans)	fMRI 7T BOLD	1000 ms (1200 ms halo)	1414	72	Gutierrez-Barragan et al., (2023) Current Biology
Nematode (C. elegans) N=10	0% iso (10 scans)	4% isoflurane (10 scans)	GCaMP6s calcium imaging	500 ms	500	120	Awal et al., (2020) Anesthesiology

TABLE S1. Overview of the six datasets included in the present study, and their key parameters. For human, macaque, marmoset, and mouse, ‘Spatial coverage’ indicates the number of cortical regions. For the nematode, it indicates the number of head neurons. MION, monocrystalline iron oxide nanoparticle contrast agent.

Feature name	Category	Description
mode_5	Distribution	5-bin histogram mode
mode_10	Distribution	10-bin histogram mode
outlier_timing_pos	Outlier	Timing of positive extreme event
outlier_timing_neg	Outlier	Timing of negative extreme event
acf_timescale	Autocorrelation	First $1/e$ crossing of the ACF
acf_first_min	Autocorrelation	First minimum of the ACF
low_freq_power	Spectrum	Power in the lowest 20% frequencies
centroid_freq	Spectrum	Centroid frequency
forecast_error	Forecasting	Error of 3-point rolling mean forecast
whiten_timescale	Forecasting	Change in autocorrelation timescale after incremental differencing
High fluctuation	Other	Proportion of high incremental changes in the series
stretch_high	Symbolic	Longest stretch of above-mean values
stretch_decreasing	Symbolic	Longest stretch of decreasing values
entropy_pairs	Symbolic	Entropy of successive two-symbol motifs in the symbolized series
ami2	Autocorrelation	Histogram-based automutual information (lag 2, 5 bins)
time_revers	Correlation	Time reversibility
ami_timescale	Autocorrelation	First minimum of the AMI function
transition_variance	Symbolic	Transition matrix column variance
periodicity	Other (periodicity)	Wang's periodicity metric
embedding_dist	Correlation	Goodness of exponential fit to embedding distance distribution
rs_range	Self-affine scaling	Rescaled range fluctuation analysis (low-scale scaling)
dfa	Self-affine scaling	Detrended fluctuation analysis (low-scale scaling)

TABLE S2. Representative subset of dynamical features from *catch22*, along with broad category assignment. Note that *ami2* (full feature ID: $CO\_HistogramAMI\_even\_2\_5$) was not included in the present work, as it failed to pass the pre-filtering stage.

Gene	Full name
ADRA1A	Adrenergic receptor, α_{1A}
ADRA1D	Adrenergic receptor, α_{1D}
ADRA2A	Adrenergic receptor, α_{2A}
ADRB1	Adrenergic receptor, β_1
CALB1	Calbindin 1
CALB2	Calbindin 2 (calretinin)
CHRM1	Cholinergic receptor, muscarinic 1
CHRM2	Cholinergic receptor, muscarinic 2
CHRM4	Cholinergic receptor, muscarinic 4
CHRM5	Cholinergic receptor, muscarinic 5
CHRNA4	Nicotinic acetylcholine receptor subunit α_4
CHRNB2	Nicotinic acetylcholine receptor subunit β_2
CNR1	Cannabinoid receptor 1
DRD1	Dopamine receptor D1
DRD2	Dopamine receptor D2
DRD4	Dopamine receptor D4
GABBR2	Gamma-aminobutyric acid receptor, type B, subunit 2
GABRA1	Gamma-aminobutyric acid receptor subunit α_1
GABRA2	Gamma-aminobutyric acid receptor subunit α_2
GABRA3	Gamma-aminobutyric acid receptor subunit α_3
GABRA4	Gamma-aminobutyric acid receptor subunit α_4
GABRA5	Gamma-aminobutyric acid receptor subunit α_5
GABRB1	Gamma-aminobutyric acid receptor subunit β_1
GABRB2	Gamma-aminobutyric acid receptor subunit β_2
GABRB3	Gamma-aminobutyric acid receptor subunit β_3
GABRE	Gamma-aminobutyric acid receptor subunit ε
GABRG1	Gamma-aminobutyric acid receptor subunit γ_1
GABRG2	Gamma-aminobutyric acid receptor subunit γ_2
GABRG3	Gamma-aminobutyric acid receptor subunit γ_3
GALR1	Galanin receptor 1
GRIA1	Glutamate receptor, ionotropic, AMPA 1
GRIA2	Glutamate receptor, ionotropic, AMPA 2
GRIA3	Glutamate receptor, ionotropic, AMPA 3
GRIA4	Glutamate receptor, ionotropic, AMPA 4
GRIK1	Glutamate receptor, ionotropic, kainate 1
GRIK2	Glutamate receptor, ionotropic, kainate 2
GRIK3	Glutamate receptor, ionotropic, kainate 3
GRIK4	Glutamate receptor, ionotropic, kainate 4
GRIK5	Glutamate receptor, ionotropic, kainate 5
GRIN1	Glutamate receptor, ionotropic, NMDA 1
GRIN2A	Glutamate receptor, ionotropic, NMDA 2A
GRIN2B	Glutamate receptor, ionotropic, NMDA 2B
GRIN2C	Glutamate receptor, ionotropic, NMDA 2C
GRIN2D	Glutamate receptor, ionotropic, NMDA 2D
GRIN3A	Glutamate receptor, ionotropic, NMDA 3A
GRM1	Glutamate receptor, metabotropic 1
GRM2	Glutamate receptor, metabotropic 2
GRM3	Glutamate receptor, metabotropic 3
GRM4	Glutamate receptor, metabotropic 4
GRM5	Glutamate receptor, metabotropic 5

TABLE S3. Complete list of brain-related genes included in the present study (1/2).

Gene	Full name
HCN1	Hyperpolarization-activated cyclic nucleotide-gated channel 1
HCRTR1	Hypocretin receptor 1
HDC	Histidine decarboxylase
HRH1	Histamine receptor H1
HRH2	Histamine receptor H2
HRH3	Histamine receptor H3
HTR1A	Serotonin receptor 1A
HTR2C	Serotonin receptor 2C
HTR3B	Serotonin receptor 3B
HTR4	Serotonin receptor 4
KCNK2	Potassium channel subfamily K member 2
MBP	Myelin basic protein
MCHR1	Melanin-concentrating hormone receptor 1
NPY1R	Neuropeptide Y receptor Y1
NTSR1	Neurotensin receptor 1
OPRK1	Opioid receptor, kappa 1
OPRL1	Opioid receptor-like 1
OPRM1	Opioid receptor, mu 1
OXTR	Oxytocin receptor
P2RY12	P2Y purinergic receptor 12
P2RY14	P2Y purinergic receptor 14
P2RY6	P2Y purinergic receptor 6
PLEKHB1	Pleckstrin homology domain containing B1
PVALB	Parvalbumin
SLC6A3	Dopamine transporter
SSTR2	Somatostatin receptor 2
SSTR4	Somatostatin receptor 4
STX1A	Syntaxin 1A
VIP	Vasoactive intestinal peptide
VIPR2	Vasoactive intestinal peptide receptor 2
SST	Somatostatin

TABLE S4. Complete list of brain-related genes included in the present study (2/2).

Parameter	Score Range	Description
Exploration of the surrounding world	0 to 2	• 0 = Total absence<br>• 1 = Small search of external clues<br>• 2 = Total investigation of the environment (e.g., head orientation to a sound)
Spontaneous movements	0 to 2	• 0 = Total absence<br>• 1 = Small torso and/or limb movement<br>• 2 = Large torso and/or limb movement
Shaking / prodding	0 to 2	• 0 = Total absence<br>• 1 = Small body movement<br>• 2 = Large body movement
Toe pinch	0 to 2	• 0 = Total absence<br>• 1 = Small reflex (weak body movement, eye blinking, or cardiac rate change)<br>• 2 = Clear reaction (strong body movement, eye blinking or opening, and cardiac rate change)
Eyes opening	0 to 2	• 0 = Total absence<br>• 1 = Small blinks or eye movements<br>• 2 = Full eye opening
Corneal reflex	0 to 1	• 0 = Absent<br>• 1 = Present

TABLE S5. Scoring criteria for behavioral assessment.

**Parameter**	**Symbol**	**Value**
External current	I_0	0.382 nA
Excitatory scaling factor for I_0	W_E	1
Inhibitory scaling factor for I_0	W_I	0.7
Local excitatory recurrence	w_+	1.4
Excitatory synaptic coupling	J_{NMDA}	0.15 nA
Threshold for $F(I_n^{(E)})$	$I_{\text{thr}}^{(E)}$	0.403 nA
Threshold for $F(I_n^{(I)})$	$I_{\text{thr}}^{(I)}$	0.288 nA
Gain factor of $F(I_n^{(E)})$	g_E	310 nC^{-1}
Gain factor of $F(I_n^{(I)})$	g_I	615 nC^{-1}
Shape of $F(I_n^{(E)})$ around $I_{\text{thr}}^{(E)}$	d_E	0.16 s
Shape of $F(I_n^{(I)})$ around $I_{\text{thr}}^{(I)}$	d_I	0.087 s
Excitatory kinetic parameter	γ	0.641
Amplitude of uncorrelated Gaussian noise v_n	σ	0.01 nA
Time constant of NMDA	τ_{NMDA}	100 ms
Time constant of GABA	τ_{GABA_A}	10 ms
Neuromodulatory scaling factor	g_n^{NM}	1

TABLE S6. Fixed parameters of the dynamic mean field model and their values.
